# Epoxy reinforced with mesoporous hydrotalcite for high-voltage insulation application

**DOI:** 10.1039/d6ra07272k

**Published:** 2026-09-24

**Authors:** Mahmoud Ezzat, Abdelrahman Said, Musa A. Said, Hany M. Abd El-Lateef, Mousa A. Abd-Allah, S. M. A. El-Gamal, M. Ramadan

**Affiliations:** a Department of Electrical Engineering, Faculty of Engineering at Shoubra, Benha University Cairo 11672 Egypt abdelrahman.ghoniem@feng.bu.edu.eg; b Faculty of Computer Science, Benha National University (BNU) Al Obour Egypt abdelrahman.ghoniem@cs.bnu.edu.eg; c Department of Chemistry, Faculty of Science, Islamic University of Madinah Madinah 42351 Saudi Arabia mssaeed@iu.edu.sa; d Department of Chemistry, College of Science, King Faisal University Al-Ahsa 31982 Saudi Arabia hmahmed@kfu.edu.sa; e Chemistry Department, Faculty of Science, Ain Shams University Cairo 11566 Egypt M.Ramadan@Sci.asu.edu.eg

## Abstract

Epoxy-based solid insulation is widely used in high-voltage systems; however, its breakdown reliability and thermal endurance remain critical limitations under coupled electrothermal stresses. This study investigates mesoporous Mg/Al hydrotalcite as a multifunctional nanofiller for enhancing the dielectric, breakdown, and thermal performance of epoxy insulation. Mesoporous Mg/Al-LDH nanoparticles were synthesized *via* a co-precipitation route and functionalized with 3-aminopropyltriethoxysilane (APTES) to improve filler dispersion and interfacial compatibility with epoxy. XRD, TEM/SAED, BET/BJH, and FTIR analyses confirmed the successful formation of layered hydrotalcite nanosheets with mesoporous characteristics and effective surface functionalization. The functionalized nanoparticles were incorporated into epoxy at loadings of 1–7 wt% and evaluated using broadband dielectric spectroscopy, AC breakdown testing with Weibull statistical analysis, SEM/EDX mapping, TGA, DSC, and compressive strength. The results showed that Mg/Al-LDH incorporation maintained a moderate permittivity variation and a dielectric loss tangent below 0.01 at 50 Hz. The optimum 5 wt% nanocomposite achieved an AC breakdown strength of 36.75 kV mm^−1^, corresponding to a 22.2% improvement over neat epoxy, together with enhanced Weibull reliability. Thermal analysis showed that the degradation onset temperature increased from 341 °C to 357 °C, and the glass transition temperature improved by 22%. Compression testing showed an increase in the maximum recorded compressive stress from 103.9 MPa for neat epoxy to 128.7 MPa for the 5 wt% nanocomposite, corresponding to approximately 23.9%; however, the different deformation and failure responses limit direct comparison of compressive strength. The findings demonstrate that APTES-functionalized mesoporous Mg/Al-LDH is a promising alternative to conventional oxide nanofillers for high-performance GIS/GITL insulation applications.

## Abstract 2

Controlling the loading and surface functionalization of mesoporous Mg/Al-LDH tunes the epoxy–filler interface, thereby enhancing dielectric breakdown reliability, thermal stability, and compressive strength.

## Graphical abstract

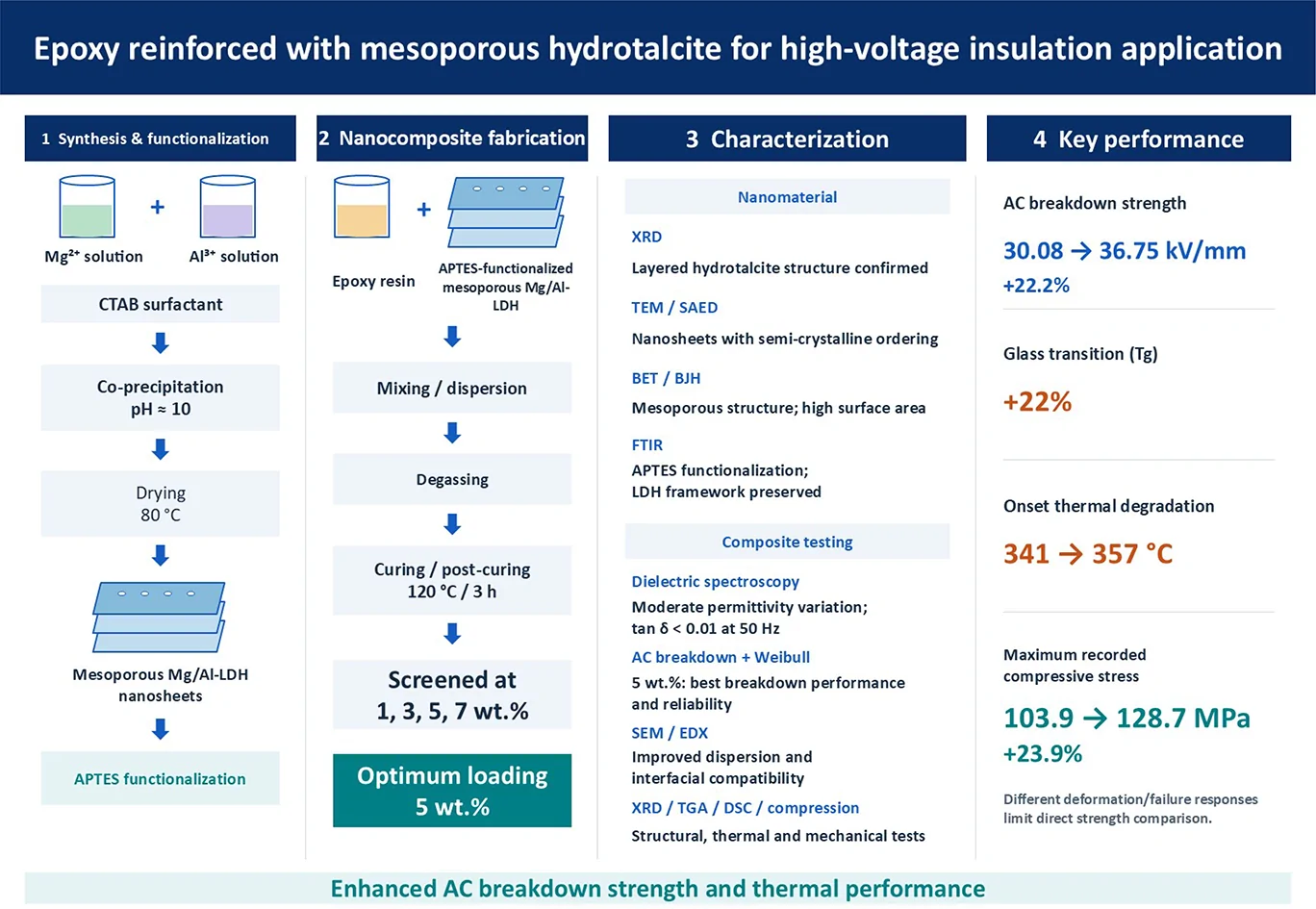

## 1 Introduction

Epoxy resin is one of the most widely used polymeric insulating materials in high-voltage engineering owing to its excellent dielectric strength, mechanical rigidity, versatility in molding, and long-term chemical stability. Its ability to form robust interfaces with metallic inserts, combined with its dimensional stability and ease of casting into complex shapes, has made epoxy a cornerstone material in numerous insulation systems.^1,2^ However, neat epoxy is not free from limitations; under high electrical stress, it suffers from space charge accumulation, interfacial polarization, restricted thermal endurance, relatively low glass transition temperature (*T*_g_), and susceptibility to electrical aging. These intrinsic constraints motivate the ongoing search for modified epoxy systems with superior electrical, thermal, and physicochemical performances suitable for next-generation power equipment.^3–5^

Among the wide range of epoxy applications, one of the most demanding and safety-critical environments is found in Gas-Insulated Substations (GIS) and Gas-Insulated Transmission Lines (GITL).^6^ In these systems, epoxy spacers serve as both mechanical supports and key dielectric components, forming an interface between metallic conductors and the surrounding SF_6_ or SF_6_-based gas mixtures. This gas–solid interface strongly governs the local field distribution, insulation reliability, and long-term aging behavior under power frequency, DC, and transient overvoltage.^2,6^ As GIS/GITL installations expand in modern high-voltage grids, especially in compact urban substations, the performance limitations of neat epoxy become more restrictive, highlighting the urgent need for enhanced epoxy-based insulation materials capable of withstanding severe electrical, thermal, and environmental stresses.^3,5,7^

The introduction of inorganic fillers into epoxy systems has long been explored as a strategy to enhance insulation performance. The early use of microscale fillers, such as SiO_2_ and Al_2_O_3_ primarily targeted improvements in mechanical strength, thermal conductivity, and mitigation of thermal expansion mismatch.^8–10^ Although these micro-composites provided some benefits, they did not deliver substantial gains in dielectric reliability or breakdown strength. With advances in nanotechnology, the focus has shifted toward nanoscale fillers that possess significantly higher interfacial areas and enhanced surface reactivity. Nanoparticles such as SiO_2_, Al_2_O_3_, TiO_2_, MgO, BaTiO_3_, and ZrO_2_ can modify charge-trapping dynamics, alter interfacial polarization, adjust permittivity, and influence the overall dielectric response, even at low loadings.^3,11^

However, the electrical performance of epoxy nanocomposites is highly dependent on the filler type, morphology, surface chemistry, and concentration. Several studies have shown contrasting behavior: for example, epoxy/SiO_2_ composites at 5 wt% can suppress the permittivity and loss tangent, while increasing the loading beyond 10 wt% reverses these improvements owing to agglomeration.^12,13^

In addition to the dielectric performance, thermal stability is an equally critical requirement for GIS spacers owing to conductor-spacer thermal coupling and potential local heating during operation. Neat epoxy typically begins to degrade near 300–350 °C and exhibits a glass transition temperature *T*_g_ value between 70 and 100 °C, depending on the formulation.^12^ The incorporation of nanofillers can delay the degradation onset, increase the residual mass, and elevate the *T*_g_ by restricting the polymer chain mobility.^1,12^

Recent studies have explored multifunctional insulating coatings, sheet-like fillers, including Ag/Cu-decorated boron nitride in PDMS, and silver@siloxane/graphene in epoxy.^14–16^ These systems highlight approaches to combining thermal transport with electrical insulation through filler design and surface modification. However, differences in polymer matrices and measured properties limit direct comparison with high-voltage epoxy insulation. Complementary evaluation of relative permittivity, dielectric loss, and AC breakdown strength is therefore needed, motivating the present investigation of Mg/Al-LDH/epoxy nanocomposites.

Layered double hydroxides (LDHs) have recently emerged as a promising class of nanofillers due to their two-dimensional lamellar morphology, rich surface chemistry, and tunable composition.^17^ LDH structures can create interfacial regions that strongly influence charge trapping, dipolar alignment, thermal decomposition behavior, and field distribution.^1,18^ Their ability to modulate both shallow and deep traps, combined with their high aspect ratio and inherent thermal stability, positions LDHs as multifunctional additives in polymer-based insulation. Experimental and computational studies have demonstrated that LDH-based epoxy nanocomposites exhibit suppressed surface charge accumulation, improved flashover resistance, reduced field distortion, and enhanced dielectric reliability in GIS environments.^2^

Recent studies have demonstrated the multifunctional role of LDH-based fillers in different polymer insulation systems. In XLPE, hybrid LDH incorporation has been reported to enhance dielectric breakdown strength and partial discharge resistance, with the breakdown strength increasing by nearly 16% and the partial discharge magnitude decreasing by about 48%.^17^ In PVDF-based composites, Mg/Al-LDH nanosheets improved dielectric breakdown strength by approximately 33% and enhanced charge discharge efficiency by about 18%.^18^ For PVC, Zn/Al–NO_3_-LDH produced a progressive increase in permittivity proportional to the LDH loading.^19^ Similarly, Zn/Al-LDH incorporation into PVA increased the permittivity from 35 for neat PVA to 334 and extended the thermal resistance to 250 °C.^20^ In epoxy-based systems, Mg/Al-LDH was reported to improve Young's modulus by approximately 20%,^21^ while Zn/Al-LDH/epoxy nanocomposites enhanced surface resistivity by approximately 53%, accelerated surface charge decay by about 20%, increased flashover voltage by 9.3%, and reduced electric-field stress at the triple-junction region by 14.7%.^2^ These findings confirm the broad potential of LDH materials as multifunctional modifiers for polymer insulation systems.

Our previous work investigated Zn/Al-LDH/epoxy nanocomposites incorporating rod-like fillers with a Zn/Al molar ratio of 2 : 1 and reported a 24% improvement in breakdown strength.^1^ The present study extends this investigation to mesoporous Mg/Al-LDH with a Mg/Al molar ratio of 3 : 1 and a layered nanosheet morphology. The two filler systems therefore differ in divalent-metal identity, metal-ion ratio, and particle morphology, rather than filler composition alone. These differences provide a basis for investigating how a chemically and morphologically distinct LDH system influences epoxy insulation performance, because filler chemistry and geometry can affect polymer–filler interactions and the resulting interfacial regions. Accordingly, the contribution of this work is to evaluate the combined dielectric, AC breakdown, thermal, and compressive response of epoxy containing APTES-functionalized Mg/Al-LDH nanosheets, rather than to establish a higher breakdown-strength enhancement than that previously obtained with Zn/Al-LDH. Filler loadings of 1–7 wt% are examined through dielectric spectroscopy, AC breakdown testing, Weibull analysis, and structural characterization, followed by thermal and compressive assessments of the formulation exhibiting the highest mean breakdown strength. Since filler chemistry, metal-ion ratio, and morphology vary together between the two studies, their individual contributions cannot be isolated through this comparison.

## 2 Materials and methods

The epoxy used in this study was KEMAPOXY 150 JM (Chemicals for Modern Building International, Giza, Egypt), formulated for electrical insulation applications. Component A is a medium-viscosity bisphenol-A/epichlorohydrin-based epoxy resin with a viscosity of approximately 10 000 mPa s and an epoxy equivalent weight (EEW) of approximately 185 g/eq., whereas Component B is a low-viscosity difunctional primary amine (polyoxypropylenediamine) curing agent with a viscosity of approximately 300 mPa s and an average molecular weight of approximately 230 g mol^−1^.^22^ According to the manufacturer's technical datasheet, the epoxy system conforms to ASTM D150, ASTM D257, and ASTM D495 standards for dielectric constant, volume resistivity, and arc resistance, respectively.^23–25^ The system has a density of 1.15 ± 0.02 kg L^−1^ and was mixed at a resin-to-hardener ratio of 3 : 1 by weight. The epoxy compound exhibits a pot life of approximately 45 min, an initial setting time of 10–12 h, a final curing time of 24 h. The specifications, purity levels, and suppliers of all chemicals and raw materials used in the synthesis and fabrication processes are summarized in Table S1.

Mg/Al hydrotalcite (Mg/Al-LDH) nanoparticles were synthesized *via* a controlled co-precipitation method. A 0.5 M magnesium nitrate solution (Mg (NO_3_)_2_) was mixed with a 0.5 M aluminum chloride solution (AlCl_3_) at a Mg^2+^/Al^3+^ volumetric ratio of 3 : 1 under continuous magnetic stirring. Simultaneously, 1.5 g of cetyltrimethylammonium bromide (CTAB) was dissolved in 15 mL of deionized water at 60 °C and subsequently introduced into the mixed metal precursor solution. After 1 h of stirring, a 2 M sodium carbonate solution (Na_2_CO_3_) was added dropwise until the suspension reached pH ≈10, which promoted the formation of a brucite-like Mg/Al hydrotalcite structure. The resulting suspension was aged under stirring for an additional 1 h, then, filtered, and washed repeatedly with deionized water until a neutral pH was reached. The obtained precipitate was dried at 80 °C overnight to yield Mg/Al hydrotalcite powder, as illustrated in Fig. S1.^2^

To improve the interfacial compatibility with the epoxy matrix, the synthesized Mg/Al hydrotalcite was surface-functionalized using 3-aminopropyltriethoxysilane (APTES). Approximately 3 g of hydrotalcite powder was dispersed in 60 mL of ethanol, ultrasonicated at 70 °C for 30 min, and mechanically stirred at 500 rpm for 30 min. A 10 wt% ethanolic APTES solution was then added dropwise, and the suspension was maintained at 70 °C for 5 h to promote silane grafting onto the hydroxyl-rich hydrotalcite surface. The modified nanoparticles were subsequently filtered, rinsed with ethanol, and dried overnight at 80 °C. This treatment reduced the hydrophilicity of the filler and improved its dispersion stability and interfacial affinity within the epoxy matrix, as shown in Fig. S2.^26,27^

For clarity, a photographic illustration summarizing the different stages of Mg/Al-LDH preparation is provided in Fig. S3.

Finally, hydrotalcite/epoxy nanocomposites were prepared by combining ultrasonic dispersion with mechanical mixing to ensure homogeneous filler incorporation. Prior to mixing, the functionalized hydrotalcite was dried at 80 °C for 12 h to remove residual moisture, then dispersed in ethanol, and ultrasonicated for 1 h. The resulting suspension was gradually introduced into the epoxy resin under continuous mechanical stirring for 2 h, followed by an additional 30 min ultrasonication step to improve particle distribution and exfoliation. Ethanol was then removed at 80 °C until a constant weight was achieved. The solvent-free nanofilled epoxy was subsequently mixed with a curing agent at a resin-to-hardener ratio of 3 : 1 by weight and stirred gently for 3 min. The blend was then kept on a preheated hot plate at 40–45 °C for 10 minutes to reduce viscosity and allow entrapped bubbles to escape before casting, cast into silicone molds, and cured under ambient conditions for 24 h, followed by post-curing at 120 °C for 3 h.^2^ The cured specimens were then demolded, polished, and stored in a desiccator prior to electrical and structural characterization. A schematic illustration of the fabrication route is provided in Fig. S4. A photographic summary of the main Mg/Al-LDH/epoxy nanocomposite sample preparation steps is presented in Fig. S5.

The experimental methodology was designed as a four-stage workflow to systematically evaluate the suitability of Mg/Al hydrotalcite-reinforced epoxy nanocomposites for high-voltage insulation applications. The overall experimental framework is schematically summarized in Fig. S6.

In the first stage, the synthesized Mg/Al hydrotalcite nanoparticles were structurally characterized to confirm their morphology, crystallinity, and textural properties. High-resolution transmission electron microscopy (HR-TEM, JEOL JEM-2100 Plus, Japan) operated at 200 kV and equipped with selected area electron diffraction (SAED) was used to examine platelet morphology, particle dimensions, and local crystallographic ordering. The phase composition and structural ordering were further analyzed using X-ray diffraction (XRD, PANalytical X'Pert PRO, Netherlands) with Cu Kα radiation (*λ* = 1.5406 Å) over a 2*θ* range of 5–70° and a step size of 0.02°. The pore structure characteristics were investigated using N_2_ adsorption–desorption measurements at 77 K employing a BELSORP-mini-X analyzer (S/N: 151, Version 1.0.9.0). Prior to analysis, the samples (LDH and functionalized LDH) were degassed under vacuum (<10^−2^ mbar) at 373 K for 12 h to eliminate physically adsorbed moisture and surface contaminants while preserving the structural integrity of the LDH framework and maintaining the grafted silane functionalities of the APTES-modified sample. The specific surface area was determined using the Brunauer–Emmett–Teller (BET) method, applying a relative pressure (*P*/*P*_0_) fitting range of 0.05–0.35. The total pore volume was estimated from the amount of nitrogen adsorbed at a relative pressure close to saturation (*P*/*P*_0_ ≈ 0.99), assuming complete pore filling by liquid nitrogen. The pore size distribution was calculated from the desorption branch using the Barrett–Joyner–Halenda (BJH) model.

The chemical structure and surface functional groups of the pristine and APTES-functionalized Mg/Al-LDH nanoparticles were characterized using Fourier-transform infrared spectroscopy (FTIR, Unicam Mattson 1000). The spectra were recorded in the wavenumber range of 400–4000 cm^−1^. FTIR analysis was performed to identify the characteristic vibrational bands of the LDH structure and to evaluate the effectiveness of the surface functionalization process. The obtained spectra were used to assess the presence of silane-derived functional groups and to verify the preservation of the LDH framework following APTES modification.

In the second stage, hydrotalcite/epoxy nanocomposites were fabricated with filler loadings of 1, 3, 5, and 7 wt% to evaluate the influence of filler concentration on insulation performance. These concentrations were selected to establish a systematic screening range for identifying the optimum filler loading.

In the third stage, the electrical performance of the prepared nanocomposites was assessed through dielectric and breakdown screening. Dielectric characterization was performed using an Alpha-A High-Performance Frequency Analyzer (Concept 40, Novocontrol Technologies, Germany) over the frequency range of 10^−1^ to 10^7^ Hz, using electrodes with a diameter of 30 mm and an applied AC voltage of 1 V_rms_. Measurements were conducted at 25 °C and 50% relative humidity. The relative permittivity (*ε*_r_) and dielectric loss tangent (tan *δ*) were extracted to evaluate dielectric polarization behavior, interfacial relaxation, and energy dissipation across the investigated filler concentrations. Three separate specimens were measured for each formulation, including neat epoxy. The relative permittivity and dielectric loss tangent curves represent the mean values of these three specimens at each measured frequency.

The AC breakdown strength of the hydrotalcite/epoxy nanocomposites was evaluated using a Megger Foster 60AF/2 oil dielectric tester (UK) in accordance with ASTM D149.^28^ For each composition, 15 specimens (5 cm × 5 cm, 1 mm thickness) were tested between mushroom electrode 25 mm diameter with rounded edges inside insulating oil to suppress surface flashover and ensure bulk dielectric failure. The applied voltage was increased linearly at a rate of 500 V s^−1^ until breakdown occurred. The resulting breakdown data were analyzed using a two-parameter Weibull statistical model to determine the characteristic breakdown strength and statistical reliability of each composition. Differences in mean AC breakdown strength among the five formulations (*n* = 15 each) were assessed using one-way ANOVA followed by Tukey's HSD test at *α* = 0.05. Normality and homogeneity of variance were assessed using Shapiro–Wilk and median-centered Levene tests, respectively.

In the fourth stage, the nanocomposite composition exhibiting the highest breakdown strength was selected for advanced structural and thermal characterization to clarify the governing enhancement mechanisms. The microstructure of the optimized hydrotalcite/epoxy nanocomposite was examined using field-emission scanning electron microscopy (FESEM, Thermo Scientific Quattro S, USA) coupled with energy-dispersive X-ray spectroscopy to evaluate the filler dispersion, interfacial morphology, and elemental distribution within the epoxy matrix. Complementary X-ray diffraction measurements were also performed on the cured nanocomposite to assess structural changes following filler incorporation.

The thermal stability and transition behavior of the optimized nanocomposite were evaluated using thermogravimetric analysis (TGA) and differential scanning calorimetry (DSC) with a THEMSYS ONE thermal analyzer (SETARAM, France). The measurements were conducted from room temperature to 700 °C at a heating rate of 15 °C min^−1^ in a nitrogen atmosphere. TGA and DSC were each performed on one specimen per formulation. TGA was used to assess the thermal degradation behavior, decomposition onset, and residual char formation. The onset temperature of the main mass-loss event was determined using the instrument software from the intersection of the linear baseline with the tangent to the leading edge of the corresponding DTG peak.

DSC was used to determine the glass transition temperature (*T*_g_) and evaluate the influence of hydrotalcite incorporation on polymer chain mobility and thermal stability. The DSC thermograms reported in this study were obtained from the first heating cycle to evaluate the thermal behavior of the fabricated nanocomposites in their as-prepared state, including the glass transition temperature and relaxation enthalpy associated with the curing history of the epoxy network.

To complement the structural and thermal analyses, compression testing was conducted to assess the mechanical integrity of the optimized nanocomposite under compressive loading, which is relevant to handling, installation, and service conditions of epoxy-based GIS/GITL spacer materials. The neat epoxy and the optimized 5 wt% Mg/Al-LDH epoxy nanocomposite were tested according to ASTM D695.^29^ Five specimens were prepared for each formulation with the nominal dimensions shown in Fig. S7 (25.4 mm × 12.7 mm × 12.7 mm). The tests were performed using a CONTROLS hydraulic compression testing machine equipped with a datamatic digital control/readout unit (CONTROLS, Milano, Italy) with flat parallel compression platens under monotonic displacement-controlled loading of 1.3 mm min^−1^. The maximum compressive stress was calculated from the maximum recorded load and the initial loaded cross-sectional area of each specimen. After compression, the tested specimens were photographed to examine the post-test deformation/failure morphology. Also, results are reported as the mean ± standard deviation of five specimens per formulation. The difference in mean maximum recorded compressive stress between the two formulations was assessed using a two-sided Welch's independent-samples *t*-test at a significance level of 0.05.

## 3 Results and discussion

### 3.1 Characterization of hydrotalcite (HTs)

The synthesized Mg/Al–CO_3_ LDH, both before and after APTES functionalization, was comprehensively characterized using XRD, FTIR, thermogravimetric analysis (TGA), N_2_ adsorption–desorption (BET/BJH), and HR-TEM/SAED to investigate its crystal structure, surface chemistry, thermal stability, textural properties, and morphological features.

Fig. 1(a) presents the XRD patterns of the pristine LDH and APTES-functionalized LDH samples. The diffraction profiles of both materials are characteristic of hydrotalcite-like Mg–Al carbonate LDHs and can be indexed to the hexagonal phases Mg_6_Al_2_CO_3_(OH)_16_·4H_2_O (PDF no. 00-014-0525) and Mg_0.75_Al_0.25_(OH)_2_(CO_3_)_0.125_ (PDF no. 01-088-9171). The pristine LDH exhibits well-defined reflections at 2*θ* ≈ 11.53° (003), 23.29° (006), 34.69° (012), 39.27° (015), 46.55° (018), 60.79° (110), and 61.83° (113), indexed according to the *R*3̄*m* hydrotalcite structure (PDF no. 00-014-0525). The corresponding interplanar spacings (*d*-values) are approximately 7.67, 3.82, 2.58, 2.29, 1.95, 1.52, and 1.50 Å, respectively. Furthermore, the diffraction data confirm the predominant formation of hydrotalcite-type Mg–Al–CO_3_ LDH, with no detectable evidence of secondary crystalline phases. Quantitative analysis using Match! software revealed an average crystallite size of approximately 8.95 nm and a crystallinity degree of 57.78%, confirming the nanocrystalline and semi-crystalline nature of the synthesized LDH.^30–32^ A schematic representation of the synthesized hydrotalcite structure is presented in Fig. 1(b). Following APTES functionalization, the characteristic LDH reflections remained at nearly identical 2*θ* positions, indicating that the hydrotalcite-like layered structure was preserved. In particular, the basal (003) reflection remained essentially unchanged, corresponding to an interlayer spacing (d003) of approximately 7.67 Å. The absence of a significant shift in the basal spacing suggests that no interlayer expansion occurred during the grafting process. This finding indicates that APTES molecules were predominantly attached to the external surface and edge hydroxyl groups of the LDH layers rather than being intercalated into the interlayer galleries. Furthermore, the absence of additional crystalline phases confirms that the functionalization process did not alter the crystal structure of the Mg–Al–CO_3_ LDH.^33^

**Fig. 1:**
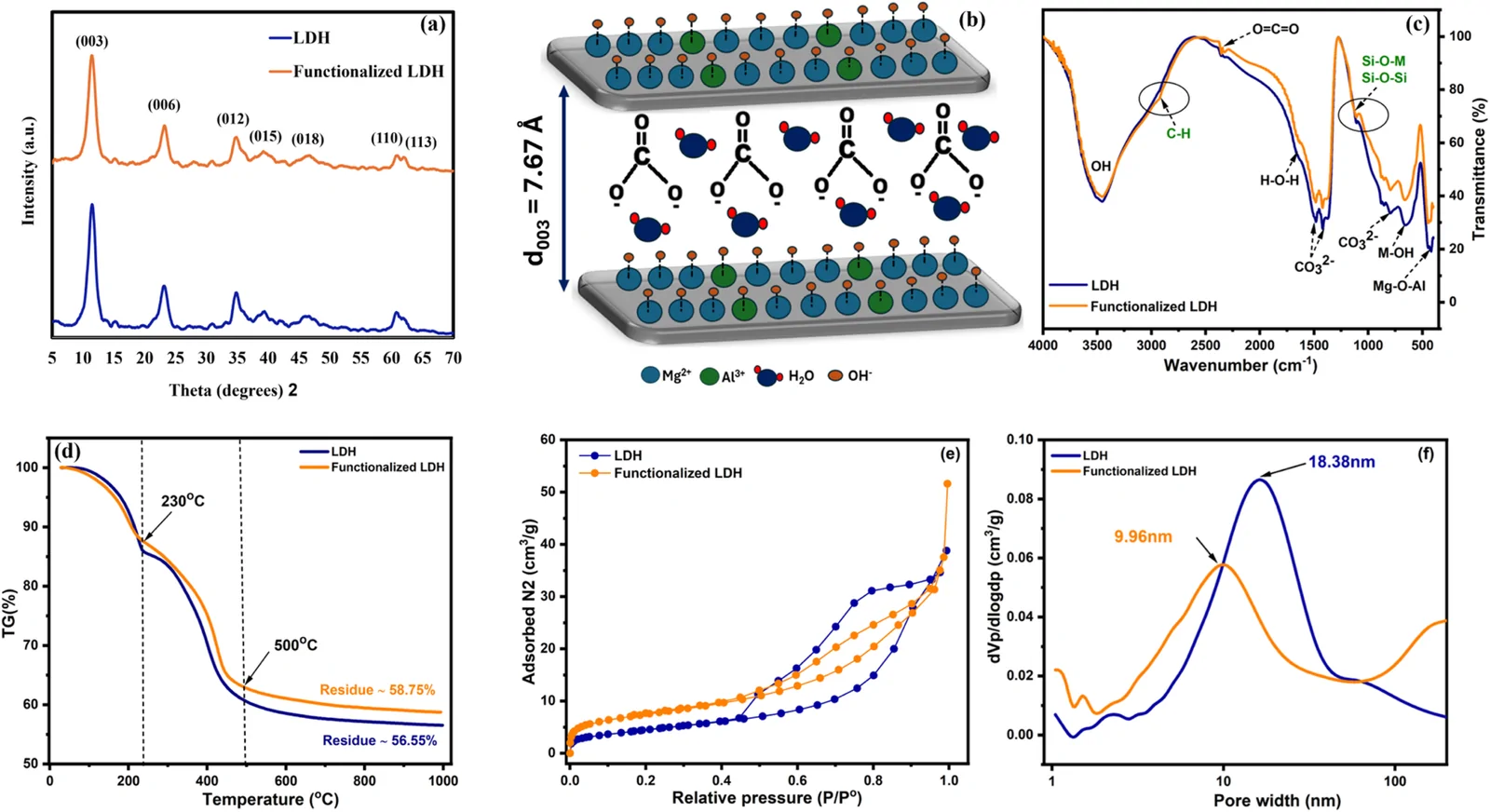
(a) XRD patterns, (b) schematic illustration of MgAl–CO_3_ LDH, (c) FTIR spectra, (d) TGA thermograms, (e) N_2_ adsorption–desorption isotherms, and (f) BJH pore size distribution curves of pristine LDH and APTES-functionalized LDH.

The FTIR spectrum of MgAl–CO_3_·OH·H_2_O LDH as shown in Fig. 1(c) displays several distinctive absorption bands that reflect its structural features. A broad and intense band in the 3100–3750 cm^−1^ region is attributed to the O–H stretching vibrations, arising from both the hydroxyl groups within the brucite-like layers and the interlayer water molecules. A weaker band near 1650 cm^−1^ corresponds to the bending vibration of H–O–H, indicating the presence of interlayer water. The existence of carbonate anions in the interlayer space is confirmed by strong bands at 1425 and 1480 cm^−1^, which are assigned to the asymmetric stretching modes of CO_3_^2−^. A weak absorption band observed at 2300–2400 cm^−1^ due to the asymmetric stretching of atmospheric CO_2_ which adsorbed on the surface of LDH during handing. Additional weaker absorption around 800 cm^−1^ is associated with the bending vibrations of carbonate ions. In the low-wavenumber region, the bands observed at approximately 660 cm^−1^ and 420 cm^−1^ are attributed to metal–oxygen vibrations, corresponding to stretching (Mg–OH or Al–OH) and bending (Mg–O–Al) modes, respectively. Altogether, these spectral features verify the characteristic structure of the synthesized hydrotalcite material.^34^ After functionalization with APTES, the FTIR spectrum of LDH shows notable changes that confirm the introduction of organic silane groups onto the surface. In addition to the characteristic LDH bands, new absorption bands appear at ∼2900–2990 cm^−1^, attributed to the C–H stretching vibrations of –CH_2_ groups from the propyl chain of APTES.^35^ A significant increase in intensity at the 1080–1150 cm^−1^ region is assigned to Si–O–Si and Si–O–M (M = Mg or Al) stretching vibrations, indicating the formation of covalent bonds between hydrolyzed silane and the LDH surface.^36^ Further supporting the grafting process. Importantly, the carbonate bands near 1425–1480 cm^−1^ remain present, confirming that the LDH layered structure is preserved after modification. The decrease in peak intensities after APTES functionalization can be attributed to the formation of an organic silane layer on the LDH surface, which partially masks the characteristic vibrations of the inorganic framework, reduces the effective interaction of infrared radiation with LDH functional groups, and introduces structural disorder and hydrogen-bonding interactions that lead to reduced absorption-band intensity.^37^ These spectral changes collectively demonstrate the successful functionalization of LDH with APTES.

Fig. 1(d) presents the TGA curves of pristine Mg/Al-LDH (navy) and APTES-functionalized LDH (orange), both exhibiting a characteristic three-stage thermal decomposition behavior. The initial mass loss up to 230 °C is attributed to the removal of physically adsorbed and interlayer water molecules. The main decomposition stage occurs between 230 and 500 °C, corresponding to the dehydroxylation of the brucite-like layers, decomposition of interlayer carbonate ions, and collapse of the LDH structure.^38^ In the functionalized LDH, this stage also includes the degradation of grafted APTES molecules. Despite the presence of organic silane groups, the functionalized LDH shows slightly improved thermal stability and consistently higher mass retention throughout the heating process. Above 500 °C, only minor weight losses are observed due to the complete decarbonation in addition to the formation of thermally stable Mg–Al mixed oxides. The final residue increased from 56.55 wt% for pristine LDH to 58.75 wt% for functionalized LDH, confirming successful silane grafting and suggesting the formation of thermally stable silicon-containing species (Si–O–Mg or Si–O–Al) that enhance the thermal resistance of the modified nanoparticles.^39,40^

As clarified in Fig. 1(e and f), the N_2_ adsorption–desorption isotherms (Fig. 1(e)) and BJH-pore size distributions (Fig. 1(f)) of both pristine LDH and APTES-functionalized LDH exhibit characteristics of type IV isotherms with H3-type hysteresis loops, indicating the predominance of mesoporous structures generated by the aggregation of plate-like LDH particles.^41^ The pristine LDH showed a BET surface area of 16.89 m^2^ g^−1^, an adsorption capacity of 3.88 cm^3^ g^−1^, a total pore volume of 0.059 cm^3^ g^−1^, and a dominant pore diameter of 18.38 nm, confirming its mesoporous nature. Following APTES functionalization, the textural properties were significantly altered, with the BET surface area increasing to 26.51 m^2^ g^−1^ which falls within the range of values previously reported for Mg/Al-LDH,^42–44^ the adsorption capacity increasing to 6.09 cm^3^ g^−1^, and the total pore volume slightly increasing to 0.066 cm^3^ g^−1^. Simultaneously, the dominant pore diameter decreased to 9.96 nm, suggesting that the grafted silane molecules partially occupied the larger pores while promoting better particle dispersion and reducing agglomeration. The increase in surface area and pore volume, together with the reduction in pore size, indicates that APTES modification effectively changed the surface characteristics of LDH by introducing organosilane functionalities, resulting in a more accessible mesoporous structure with enhanced interfacial interaction potential. These findings, combined with the higher thermal residue observed in TGA, provide strong evidence that APTES was successfully incorporated onto the LDH surface and significantly improved its physicochemical properties. The increase in surface area and pore volume observed after APTES modification agrees with the mechanism proposed by Tao *et al.*,^41^ who reported that silanization can enhance the textural properties of LDHs by reducing water-associated aggregation, modifying particle packing, and generating additional mesopores through silane-induced interparticle crosslinking.

The functionalized MgAl–CO_3_ LDH exhibits the characteristic lamellar morphology of hydrotalcite-like materials, as observed in the TEM micrograph Fig. 2(a). The structure consists of ultrathin plate-like nanosheets with a highly wrinkled and partially restacked appearance, forming loosely aggregated assemblies with lateral dimensions reaching approximately 100 nm. This morphology is typical of LDHs and reflects the stacked hydroxide-layer structure, consistent with the pronounced basal reflection observed in the XRD pattern at 2*θ* ≈ 11.53°, indexed to the (003) plane. The HR-TEM image, together with the IFFT-assisted lattice analysis shown in Fig. 2(b), provides further evidence of the local crystallographic ordering within the synthesized LDH nanosheets. Although the lattice fringes are visible, they show limited continuity and slight irregularity, indicating partial structural ordering rather than a fully crystalline arrangement. The measured interplanar spacings of approximately 0.71 nm and 0.25 nm are assigned to the (003) basal plane and the (012) crystallographic plane, respectively. The spacing of 0.71 nm is close to the XRD-derived *d*-spacing of 0.767 nm for the (003) reflection, confirming the interlayer distance associated with carbonate-intercalated hydrotalcite structures. The slight difference between the HR-TEM/IFFT and XRD values may arise from local lattice distortion, nanoscale defects, and the limited coherence length of the fringes. These features are consistent with the semi-crystalline nature inferred from XRD analysis.^45–47^ The SAED pattern presented in Fig. 2(c) displays diffuse concentric rings rather than sharp discrete spots, confirming the polycrystalline nature of the synthesized LDH with randomly oriented nanoscale domains. The diffuse character of the rings indicates limited long-range crystallinity, in agreement with the moderate crystallinity value of 57.78% determined from XRD analysis. The observed diffraction rings can be indexed to crystallographic planes such as (003), (012)/(015), and (018), corresponding well with the reflections identified in the XRD pattern. These findings are in good agreement with earlier research reports^33,48^

**Fig. 2:**
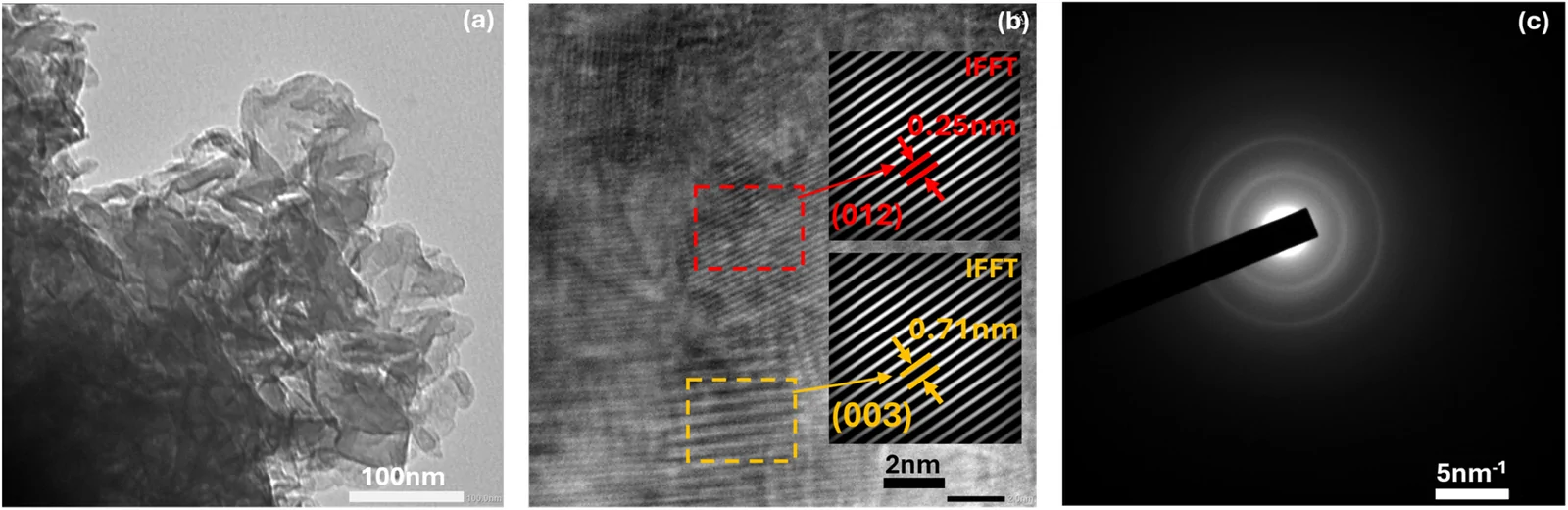
Structural and spectroscopic characterization of functionalized Mg/Al-LDH: (a) TEM, (b) HR-TEM image with IFFT and (c) SAED pattern.

### 3.2 Relative permittivity

The relative permittivity of the pristine epoxy and its nanocomposites were investigated over a wide frequency spectrum (0.1 Hz to 10 MHz), as depicted in Fig. 3(a). Furthermore, a specific analysis of the relative permittivity at the standard power operating frequency of 50 Hz is presented in Fig. 3(b).

**Fig. 3:**
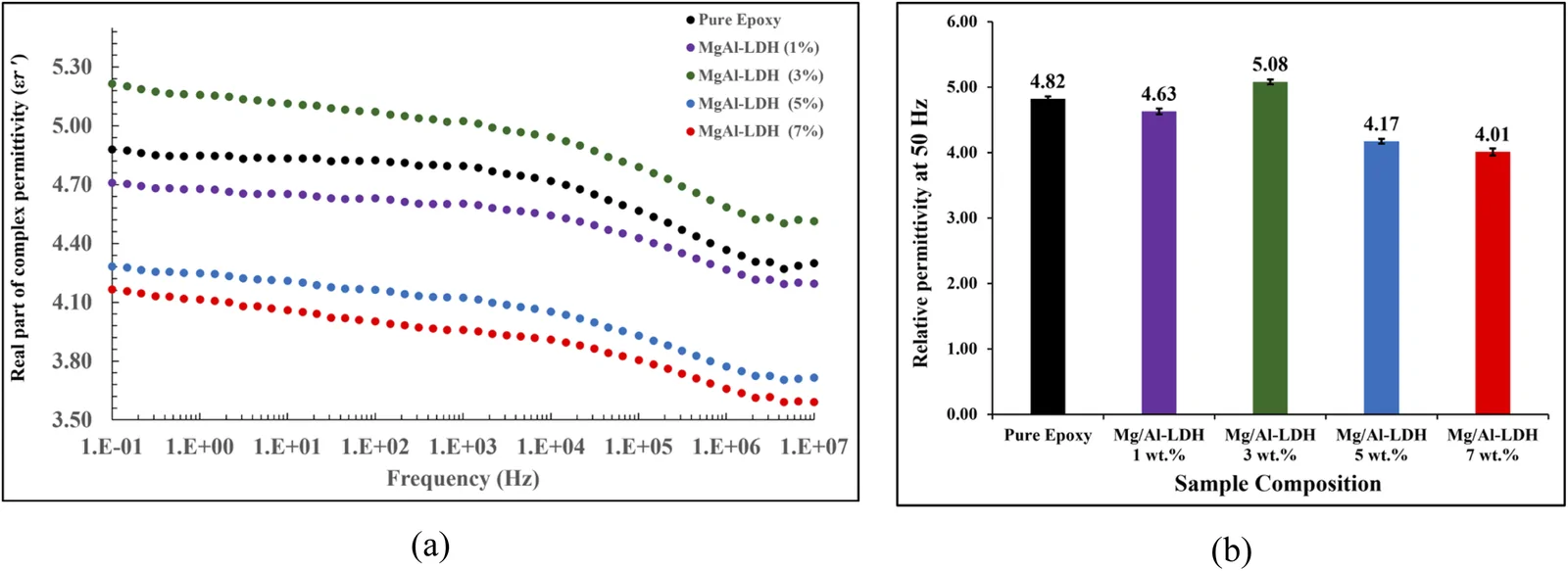
Relative permittivity of neat epoxy and Mg/Al-LDH/epoxy nanocomposites: (a) frequency dependence and (b) values at 50 Hz.

Fig. 3(a) reveals that all samples, including the pristine epoxy, exhibited a distinct dielectric relaxation phenomenon. The relative permittivity (*ε*_r_) was high at low frequencies and progressively decreased as the frequency increased. This behavior is characteristic of polymeric dielectrics and is attributed to the progressive inability of the dipolar and interfacial polarization mechanisms to follow the rapidly oscillating electric field at higher frequencies.

Notably, the relative order of the permittivity curves remained consistent across the low-to-mid frequency range. The 3 wt% LDH sample consistently showed the highest permittivity, while the 5 wt% and 7 wt% samples consistently showed the lowest. This consistent ordering suggests a filler-loading-dependent change in the dielectric response; however, the permittivity spectra alone cannot establish the degree of filler dispersion or agglomeration.^7^

For practical applications in power systems, the dielectric behavior at 50 Hz is paramount. Fig. 3(b) shows the permittivity values at this frequency, quantifying the complex, non-monotonic dependence on filler concentration.

The pristine epoxy resin establishes a baseline value of 4.82. A 1 wt% loading of Mg/Al-LDH results in a slight decrease to 4.63. At 3% loading, a significant increase was observed, with *ε*_r_ reaching a peak value of 5.08. Conversely, higher loadings of 5% and 7% lead to a sharp and continuous reduction to 4.17 and 4.01, respectively.

The non-monotonic variation in relative permittivity may reflect competing contributions from interfacial polarization and restricted polymer-chain mobility. Charge accumulation at epoxy/LDH interfaces can contribute to Maxwell–Wagner–Sillars (MWS) polarization, whereas interactions between the filler surfaces and the surrounding polymer may restrict the dipolar response of interfacial chains. The measured permittivity therefore reflects the combined influence of these contributions rather than serving as a direct measure of filler dispersion.^7,49^

At 1 wt%, the slight decrease in relative permittivity from 4.82 to 4.63 may indicate that restrictions on the polymer dipolar response outweigh the additional interfacial polarization contribution. At 3 wt%, the increase to 5.08 is consistent with a greater net contribution from polarization processes. However, this maximum does not, by itself, demonstrate optimum dispersion or exfoliation of the LDH nanosheets.

At 5 wt%, the decrease in relative permittivity to 4.17 may be associated with changes in the interfacial response and increased restrictions on polymer-chain mobility. The SEM observations discussed in Section 3.5.1 show small, localized agglomerates within the examined region of this composition. Such agglomerates may reduce the polymer-accessible filler surface relative to a fully dispersed system at the same loading, but they do not establish that the total effective interfacial area is lower than that at 3 wt%. At 7 wt%, more pronounced agglomeration is observed, which may further modify the interfacial polarization response and contribute to the lower permittivity of 4.01. These mechanisms are proposed as plausible interpretations of the measured trend; their individual contributions cannot be separated using the present permittivity measurements alone.

### 3.3 Dielectric loss

The dielectric loss tangent (tan *δ*) of the neat epoxy and hydrotalcite/epoxy nanocomposites was evaluated over the frequency range of 10^−1^ to 10^7^ Hz, with particular emphasis on the power-frequency response at 50 Hz, as shown in Fig. 4.

**Fig. 4:**
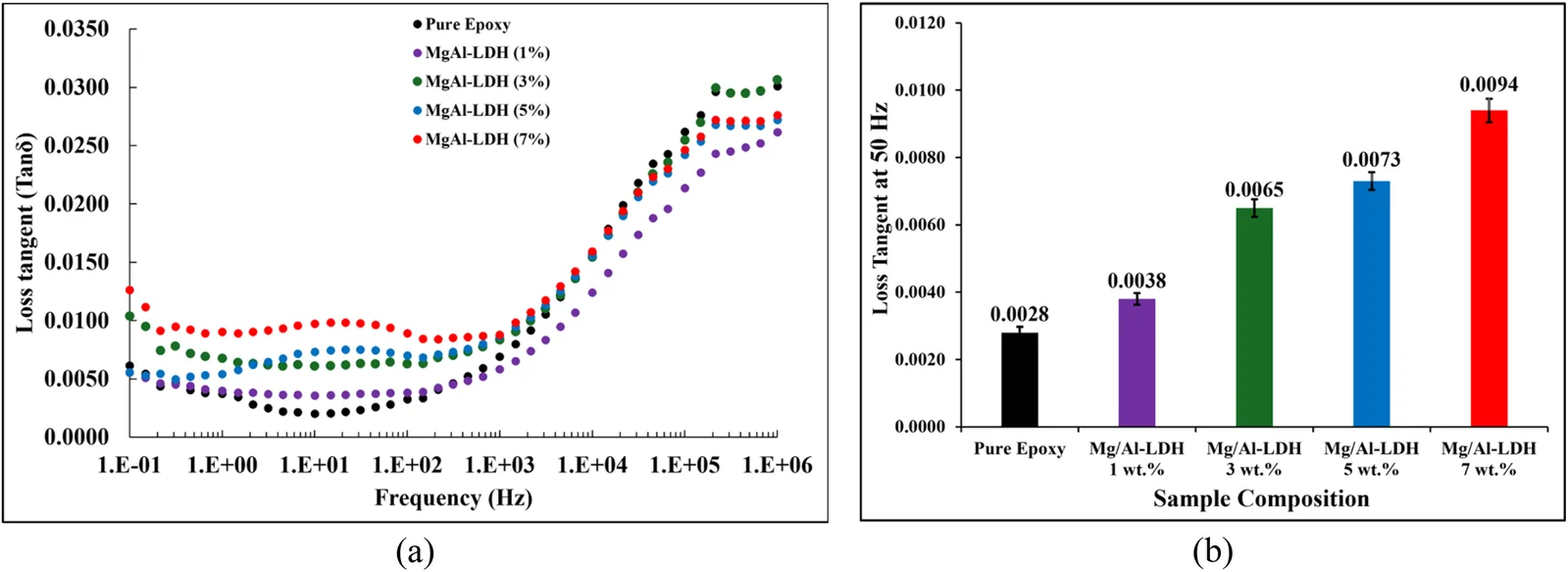
(a) Variation of tan(*δ*) over frequency, (b) tan(*δ*) at the standard power operating frequency of 50 Hz.

The frequency-dependent spectra indicate that all compositions exhibit a common dielectric relaxation feature in the high-frequency region (>10 kHz), while more pronounced differences emerge in the low-to-mid frequency range (<1 kHz), which is more relevant to insulation performance under service conditions.

As shown in Fig. 4(a), neat epoxy exhibited the lowest dielectric loss across the low-frequency region, whereas the incorporation hydrotalcite led to a progressive increase in tan *δ* with increasing filler concentration. The loss order remained consistent throughout this region and follows 7 wt% > 5 wt% > 3 wt% > 1 wt% > neat epoxy, indicating a monotonic increase in energy dissipation with filler loading. This trend is primarily attributed to the combined contribution of Maxwell–Wagner–Sillars (MWS) interfacial polarization and conduction-related losses.^49,50^

At low filler concentrations, the introduction of hydrotalcite creates additional epoxy/filler interfaces that promote interfacial charge accumulation. The repeated polarization and relaxation of these interfacial charges under alternating electric fields introduced additional dielectric dissipation, resulting in a measurable increase in tan *δ* relative to neat epoxy.

With increasing filler loading, changes in the amount and spatial distribution of epoxy/LDH interfaces may affect both interfacial relaxation and conduction-related contributions to dielectric loss. As discussed in Section 3.5.1, the examined SEM regions show small, localized agglomerates at 5 wt% and more pronounced agglomeration at 7 wt%. The limited agglomeration observed at 5 wt% indicates some microstructural heterogeneity but does not establish the formation of connected conductive pathways. At 7 wt%, the more pronounced agglomeration may contribute to a less uniform interfacial response and increased dielectric dissipation. The low-frequency upturn in tan *δ*, particularly for the 7 wt% composition, is consistent with a possible contribution from conduction-related processes in addition to interfacial relaxation.

The slight overlaps and intersections among the tan *δ* curves in the intermediate and high-frequency regions are also physically meaningful. These features arise from overlapping relaxation processes, primarily associated with the transition from interfacial (MWS) polarization to dipolar relaxation, together with minor variations in relaxation times caused by differences in filler dispersion and interfacial structure.

From an insulation engineering perspective, the loss tangent at 50 Hz is of practical importance. Diagnostic guidelines generally consider tan *δ* values below approximately 0.005 to indicate good insulation conditions, whereas substantially higher values are commonly associated with aging, contamination, or moisture-related degradation.^51^

In addition, epoxy-based insulating components tested according to IEC 60250 are typically specified to operate within a dissipation factor range of tan *δ* ≤ 0.01–0.04 at 50 Hz.^52^

In the present study, the measured loss tangent at 50 Hz ranged from 0.0028 to 0.0094 across all investigated hydrotalcite loadings, remaining within or below the acceptable range for practical epoxy-based insulation systems. This indicates that, although hydrotalcite incorporation increases dielectric dissipation, all investigated compositions retain sufficiently low loss characteristics for GIS/GITL solid insulation. Accordingly, the optimum filler concentration should be selected based on more discriminating electrical performance metrics, particularly the AC breakdown strength and statistical reliability.

### 3.4 Breakdown strength performance of epoxy nanocomposites

The AC breakdown strength of neat epoxy and hydrotalcite/epoxy nanocomposites was evaluated to assess the influence of filler loading on dielectric failure resistance. For each composition, 15 specimens were tested to ensure statistically meaningful comparison and improve confidence in the observed breakdown trends. This sample size exceeds the minimum requirement specified in ASTM D149 and provides a more reliable basis for comparative and statistical analysis.

The breakdown strength distributions, summarized in the box plot shown in Fig. 5 and statistical Table 1 collectively demonstrate a clear improvement in the breakdown strength following hydrotalcite incorporation. Neat epoxy exhibited the lowest breakdown strength, with a mean value of 30.08 kV mm^−1^, reflecting the limited ability of the unfilled matrix to suppress charge transport and delay electrical failure. In contrast, all hydrotalcite-filled nanocomposites exhibit higher breakdown strength than neat epoxy, confirming the beneficial role of Mg/Al hydrotalcite in improving dielectric robustness.

**Fig. 5:**
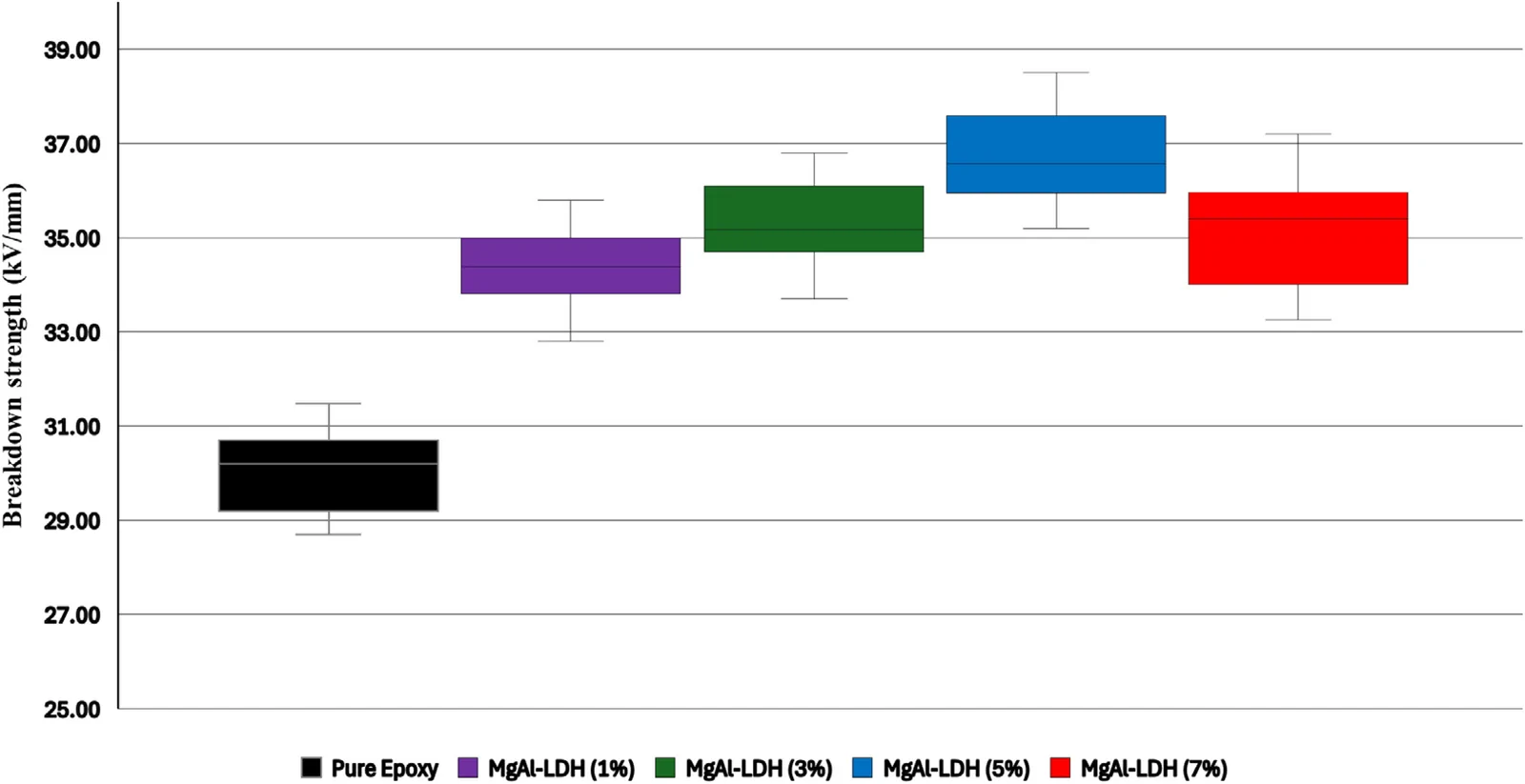
Box plots of AC breakdown strength for neat epoxy and Mg/Al-LDH/epoxy nanocomposites at different filler loadings.

**Table 1:** Statistical summary of AC breakdown strength for neat epoxy and Mg/Al-LDH/epoxy nanocomposites at different filler loadings

Composite	Min (kV mm^−1^)	Max (kV mm^−1^)	Mean ± std deviation (kV mm^−1^)
Pure epoxy	28.70	31.48	30.08 ± 0.91
Mg/Al-LDH (1 wt%)	32.80	35.80	34.33 ± 0.84
Mg/Al-LDH (3 wt%)	33.70	36.80	35.24 ± 0.94
Mg/Al-LDH (5 wt%)	35.20	38.50	36.75 ± 1.12
Mg/Al-LDH (7 wt%)	33.25	37.20	35.12 ± 1.21

As summarized in Table 1, the mean breakdown strength increases from 34.33 kV mm^−1^ at 1 wt% to 35.24 kV mm^−1^ at 3 wt%, reaching a maximum value of 36.75 kV mm^−1^ at 5 wt%. This corresponds to an improvement of approximately 22.2% relative to the neat epoxy. The 5 wt% composition also exhibited a relatively narrow box distribution in Fig. 5, indicating improved breakdown strength across the tested specimens.

One-way ANOVA indicated significant differences in mean breakdown strength among the formulations (*F*(4, 70) = 92.56, *p* < 0.001). Tukey's HSD test showed that the 5 wt% composite exceeded neat epoxy and the 1 wt% composite (both adjusted *p* < 0.001), as well as the 3 wt% (*p* = 0.00112) and 7 wt% (*p* = 0.000350) composites, supporting its selection as the optimum tested loading for mean AC breakdown strength.

The highest mean breakdown strength was obtained at 5 wt%, despite the presence of small, localized agglomerates in the examined SEM region, as discussed in Section 3.5.1. This suggests that limited agglomeration does not necessarily eliminate the beneficial effects of filler incorporation. A possible interpretation is that the epoxy/LDH interfacial regions hinder charge transport and delay the development of electrical failure, while the adverse effects of localized heterogeneity remain insufficient to outweigh these benefits. However, the formation of deep traps and the suppression of local electric-field distortion were not directly measured in this study and are therefore considered possible mechanisms rather than experimentally confirmed explanations.

At 7 wt%, the mean breakdown strength decreases to 35.12 kV mm^−1^, while the standard deviation increases from 1.12 kV mm^−1^ at 5 wt% to 1.21 kV mm^−1^. The SEM observations presented in Section 3.5.1 show more pronounced agglomeration in the examined region of the 7 wt% composite than in that of the 5 wt% composite. This morphological difference is consistent with increased microstructural heterogeneity, which may introduce local field-enhancement sites and partially offset the beneficial effects of filler incorporation.

Overall, 5 wt% is identified as the optimum loading among the investigated compositions in terms of the measured mean AC breakdown strength, rather than as a composition that maximizes every dielectric property or provides an agglomeration-free microstructure. The 3 wt% composite exhibits the highest relative permittivity, whereas the 5 wt% composite achieves the highest mean breakdown strength despite its lower permittivity and higher dielectric loss. These results indicate that the loading dependence of breakdown strength differs from that of the polarization response. The subsequent decrease in breakdown strength at 7 wt%, together with the more pronounced agglomeration observed by SEM, is consistent with a growing adverse contribution from microstructural heterogeneity. Thus, the performance maximum at 5 wt% may represent a balance between beneficial filler-related effects and the increasing influence of localized agglomeration.

#### 3.4.1 Weibull analysis for breakdown strength data

Weibull statistics were employed to further evaluate the breakdown behavior of neat epoxy and hydrotalcite/epoxy nanocomposites by accounting for the stochastic nature of dielectric failure. Unlike conventional descriptive parameters, such as the mean and standard deviation, the Weibull analysis provides a more rigorous assessment of both the breakdown strength and statistical reliability. The cumulative probability of failure is described by the two-parameter Weibull distribution in eqn (1):^53^1
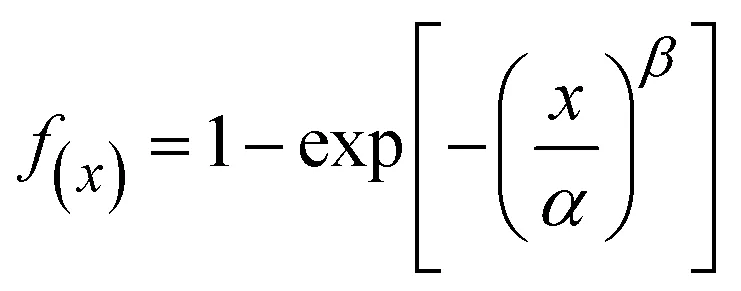
where *α* represents the characteristic breakdown strength, which represents the 63.2% probability of failure, and *β* denotes the shape parameter, which reflects the uniformity and reliability of data. For practical analysis, the Weibull distribution was transformed into its double-logarithmic linear form, as shown in eqn (2), to facilitate straightforward parameter estimation.

2 ln[−ln(1 − *f*_(*x*)_)] = *β* ln(*x*) − *β* ln(*α*)

To create this plot, the breakdown strength values were first arranged in ascending order and assigned cumulative failure probabilities using Benard's approximation, as expressed in eqn (3).^53^ This method provides an unbiased estimation of the empirical failure probability for each ranked data point, enabling an accurate linearization of the Weibull distribution for regression fitting.3
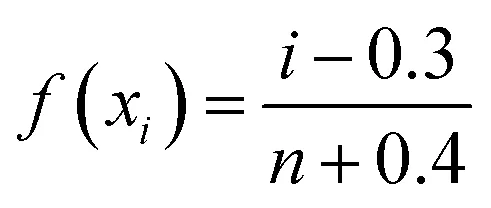
where *i* is the rank, and *n* is the number of samples. From the fitted straight line, both *α* and *β* were extracted to quantify the insulation strength and reliability. In addition, 95% confidence intervals were estimated for both *α* and *β* to quantify the parameter uncertainty. These confidence bounds were derived from the Weibull fit and used to construct the 95% confidence region of the fitted breakdown distribution.

The Weibull analysis summarized in Fig. 6 and Table 2 provides further insight into the dielectric reliability of hydrotalcite/epoxy nanocomposites relative to neat epoxy. The unfilled epoxy exhibited the lowest characteristic breakdown strength (*α* = 30.5 kV mm^−1^) with a moderate shape parameter (*β* = 39.23), indicating limited dielectric strength and moderate breakdown uniformity.

**Fig. 6:**
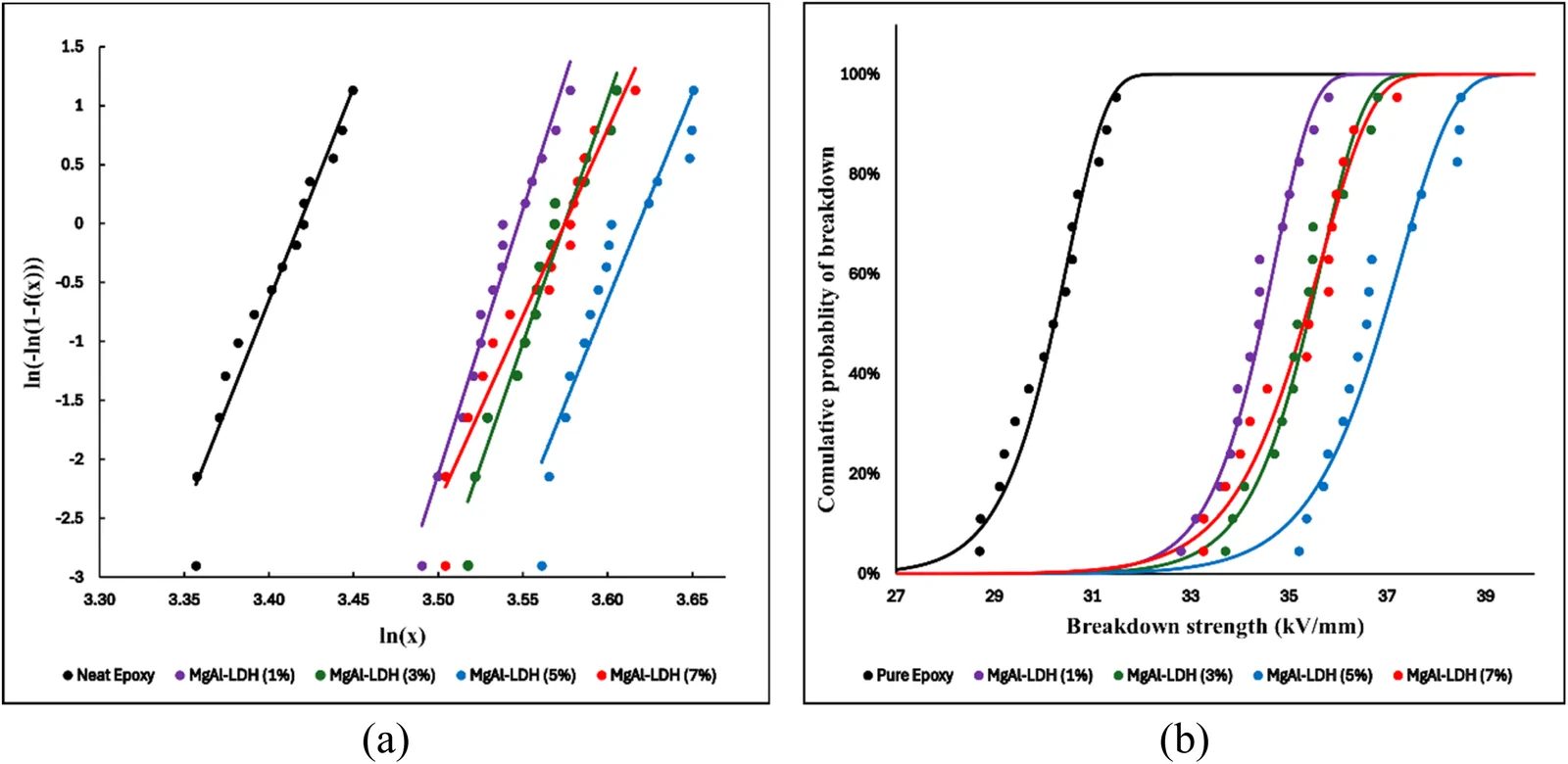
Weibull analysis of AC breakdown strength for neat epoxy and Mg/Al-LDH/epoxy nanocomposites: (a) linearized Weibull plots and (b) cumulative failure probability curves.

**Table 2:** Weibull parameters of AC breakdown strength for neat epoxy and Mg/Al-LDH/epoxy nanocomposites

Composite	Scale parameter *α* (kV mm^−1^)		Shape parameter *β*	
	*α*	95% CI	*β*	95% CI
Pure epoxy	30.5	[30.087, 30.925]	39.23	[23.569, 54.359]
Mg/Al-LDH (1 wt%)	34.73	[34.317, 35.138]	45.38	[28.028, 62.721]
Mg/Al-LDH (3 wt%)	35.68	[35.226, 36.137]	42.03	[25.960, 58.119]
Mg/Al-LDH (5 wt%)	37.29	[36.717, 37.861]	35.04	[21.607, 48.482]
Mg/Al-LDH (7 wt%)	35.67	[35.116, 36.224]	34.44	[21.009, 47.866]

Following hydrotalcite incorporation, the characteristic breakdown strength increased consistently across all investigated loadings, confirming the beneficial influence of the nanofiller on dielectric endurance. At 1 wt%, *α* increases to 34.73 kV mm^−1^, while *β* rises to 45.38, indicating not only improved breakdown strength but also enhanced statistical uniformity and reproducibility. At 3 wt%, *α* increases further to 35.68 kV mm^−1^, whereas *β* decreases slightly to 42.03, suggesting that the gain in dielectric strength is accompanied by a modest increase in microstructural heterogeneity.

The most favorable Weibull response is observed at 5 wt%, where the characteristic breakdown strength reaches its maximum value (*α* = 37.29 kV mm^−1^), corresponding to an improvement of approximately 22.3% over neat epoxy. This confirms that 5 wt% provides the most effective enhancement in the breakdown strength. Although the corresponding *β* value decreases to 35.04, it remains within an acceptable range, indicating that a substantial gain in breakdown strength is achieved with only moderate statistical dispersion. This behavior suggests that the 5 wt% provides the most favorable balance between dielectric strengthening and acceptable reliability, consistent with the breakdown trends observed in Fig. 5.

At 7 wt%, the characteristic breakdown strength decreases slightly to 35.67 kV mm^−1^ and accompanied by a further reduction in *β* to 34.44. This decline indicates that excessive filler loading introduces greater structural heterogeneity, most likely due to filler agglomeration and localized field distortion, which reduces both the dielectric strength and breakdown consistency.

Fig. 7 presents the Weibull fit of the optimum 5 wt% hydrotalcite/epoxy nanocomposite together with the corresponding 95% confidence bounds. The fitted Weibull curve closely followed the experimental cumulative failure probabilities across the full breakdown range, confirming good agreement between the measured data and fitted statistical model.

**Fig. 7:**
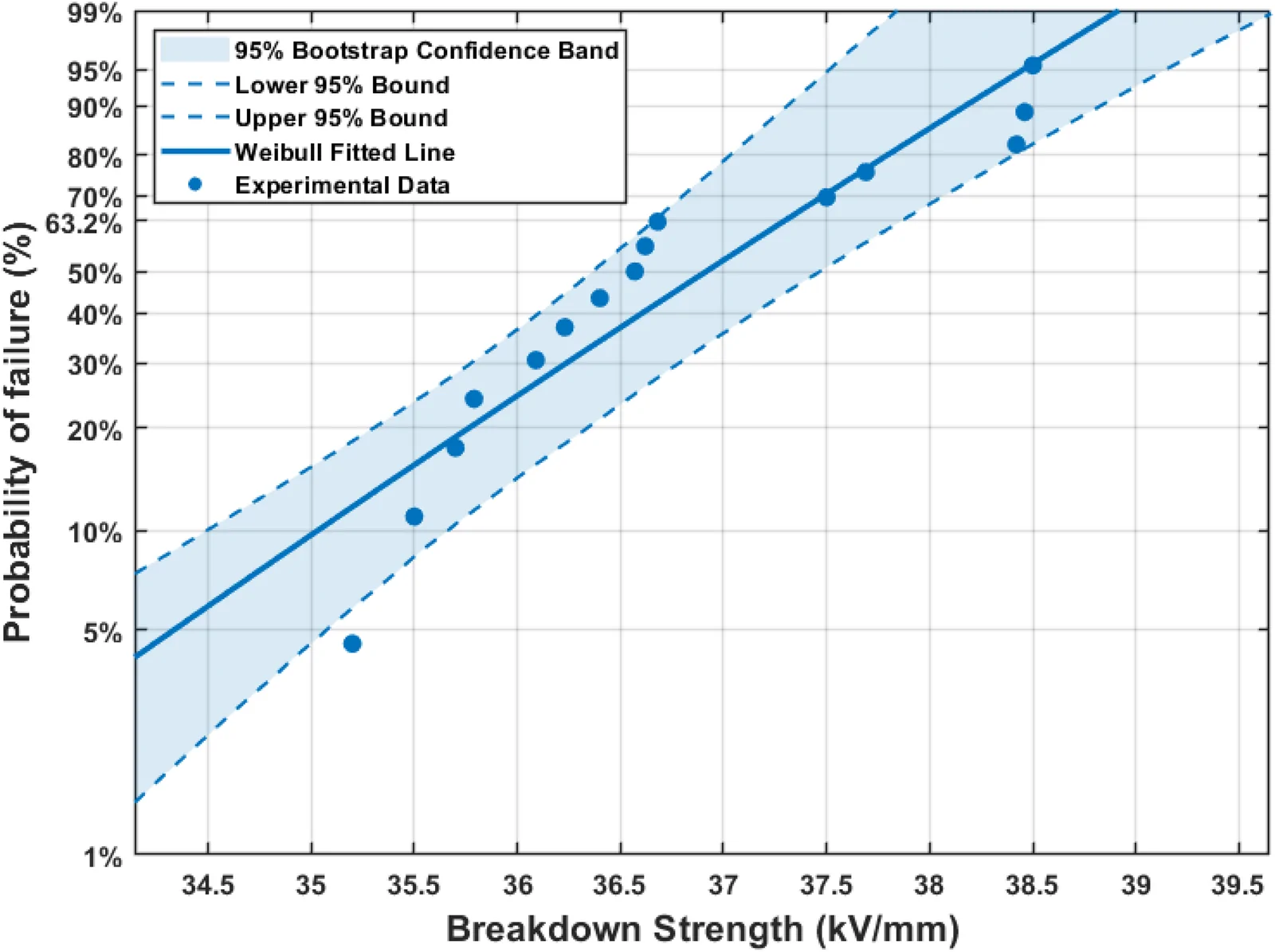
Weibull fit of AC breakdown strength for the 5 wt% Mg/Al-LDH/epoxy nanocomposite with 95% confidence bounds.

More importantly, the confidence bounds remained relatively narrow over most of the failure domain, confirming that the observed improvement in the characteristic breakdown strength was statistically reliable and not dominated by random experimental scatter. This provides additional confidence that the 5 wt% composition offers not only the highest dielectric endurance, but also a statistically robust and predictable breakdown response.

To place the obtained breakdown enhancement in a broader practical context, the performance of the present Mg/Al-LDH/epoxy nanocomposite was compared with representative epoxy-based nanocomposite insulation systems reported in the literature, as summarized in Table 3. Since the absolute breakdown strength is strongly affected by the epoxy formulation, curing chemistry, and test standard, the comparison is presented in terms of the percentage improvement relative to the corresponding neat epoxy in each study. The breakdown enhancement achieved in the present work lies within a practically relevant range for epoxy nanocomposite insulation systems. This approach provides a more objective basis for assessing the practical significance of the present improvement.

**Table 3:** Comparison of breakdown strength improvement in the present Mg/Al-LDH/epoxy nanocomposite with epoxy-based nanocomposite insulation systems reported in the literature

Epoxy nanocomposite system	Optimum filler loading	Breakdown strength improvement (%)	Ref.
EP/nano-silicides: SiO_2_, Si_3_N_4_, SiC	0.5 wt%	4.6–8.8%	54
EP/TiO_2_	1 wt%	11.7%	55
EP/γ-Al_2_O_3_	2 wt%	−22.0%	55
EP/α-Al_2_O_3_	2 wt%	∼6.7%	55
EP/POSS	2 wt%	∼15.4%	55
EP/plasma-treated Al_2_O_3_	2 wt%	∼10.5%	55
EP/microdiamond	60 wt%	42.6%	55
Mesoporous Zn/Al-LDH/EP	5 wt%	24.0%	1
Present Mg/Al-LDH/EP	5 wt%	22.2%	This study

The main objective of this work was to improve AC breakdown performance and thermal properties while maintaining moderate variations in relative permittivity and a dielectric loss tangent below 0.01 at 50 Hz across the investigated filler loadings. The 5 wt% Mg/Al-LDH/epoxy nanocomposite achieved a 22.2% enhancement in AC breakdown strength, which is comparable to or higher than several reported epoxy nanocomposite systems, while avoiding excessive filler loading.

### 3.5 Advanced characterization of epoxy nanocomposites

To clarify the mechanisms responsible for the improved dielectric and breakdown performances, the optimum hydrotalcite/epoxy nanocomposite (5 wt%) was subjected to advanced structural, morphological, and thermal characterization. X-ray diffraction (XRD), scanning electron microscopy coupled with energy-dispersive X-ray spectroscopy (SEM/EDX), thermogravimetric analysis (TGA), and differential scanning calorimetry (DSC) were employed to examine the structural organization, filler dispersion, and thermal stability of the optimized nanocomposite relative to that of the neat epoxy.

#### 3.5.1 Structural and morphological characterization

As illustrated in Fig. 8, the XRD pattern of the pristine epoxy resin exhibits two broad diffraction halos centered at 2*θ* ≈ 20–22° and 43–45°, confirming its predominantly amorphous nature arising from the lack of long-range molecular ordering in the crosslinked polymer network. Upon incorporation of 5 wt% hydrotalcite nanoparticles (HTs), the diffraction pattern of the HTs@epoxy composite displays additional reflections at 2*θ* ≈ 11.31°, 23.19°, 34.43°, 39.43°, 46.21°, 60.55°, and 61.75°, corresponding to the characteristic planes of Mg/Al-LDH (PDF no. 00-014-0525 and 01-088-9171). These reflections confirm the successful incorporation and structural preservation of the LDH nanoparticles within the epoxy matrix. Additional reflections are observed at 2*θ* ≈ 16.81°, 18.47°, 19.29°, 20.91°, 22.45°, and 24.19°. These reflections remain unassigned because the available diffraction data do not permit reliable identification of the corresponding crystalline phase or phases. They are therefore not attributed to interfacial chelation or used as evidence of increased ordering of the epoxy matrix.

**Fig. 8:**
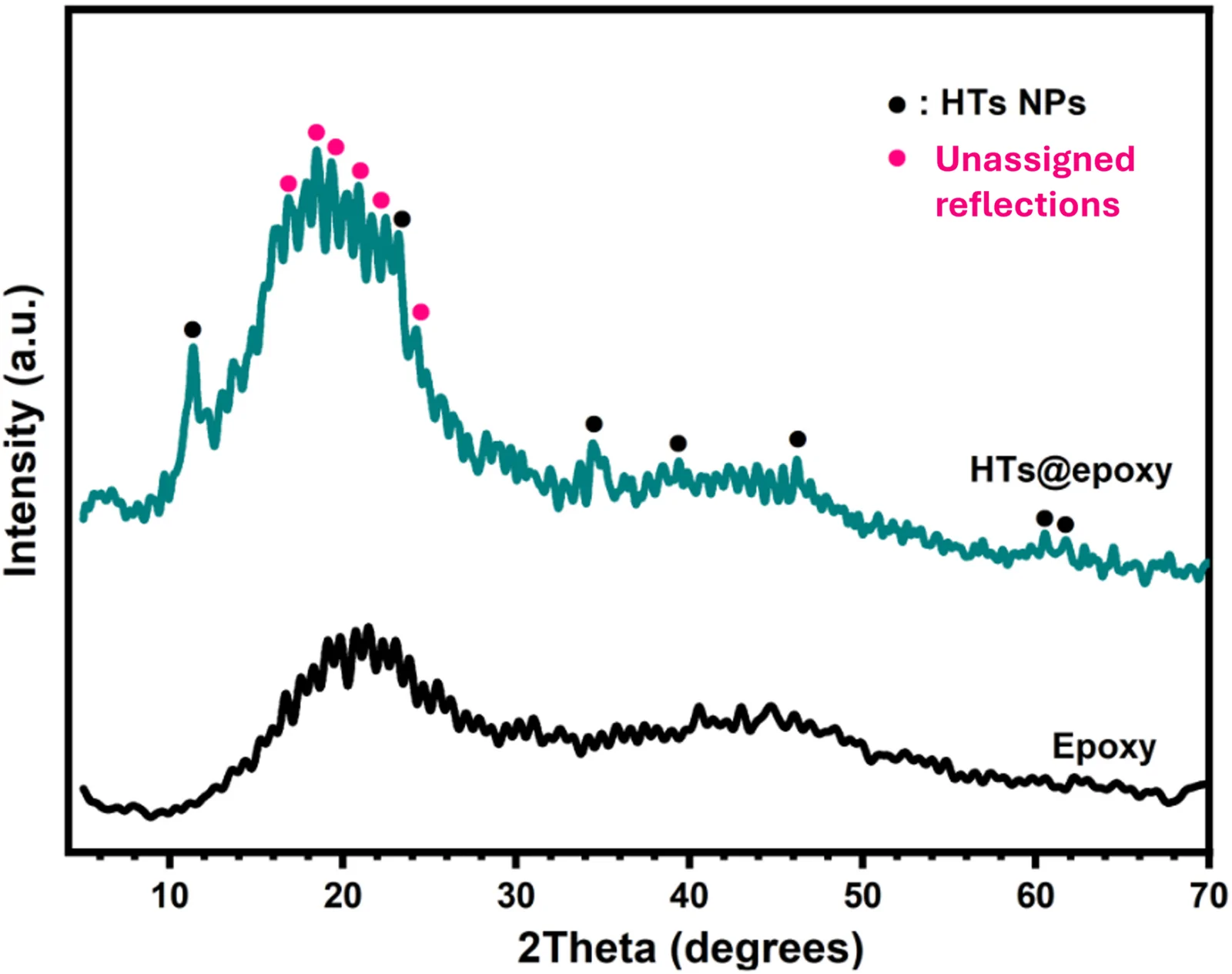
XRD patterns of neat epoxy and the 5 wt% Mg/Al-LDH/epoxy nanocomposite.

Previous studies on modified Mg/Al-LDH/epoxy and layered-silicate/epoxy nanocomposites have demonstrated restricted polymer relaxation near filler interfaces.^56,57^ Separately, local measurements in BaTiO_3_/epoxy composites have shown that filler interfaces can influence charge trapping and migration.^58^ These findings provide a literature basis for considering interfacial effects as possible contributors to the thermal and electrical responses observed here.

The morphological features of the neat epoxy and the optimized hydrotalcite/epoxy nanocomposite were further examined using SEM/EDX, as shown in Fig. 9.

**Fig. 9:**
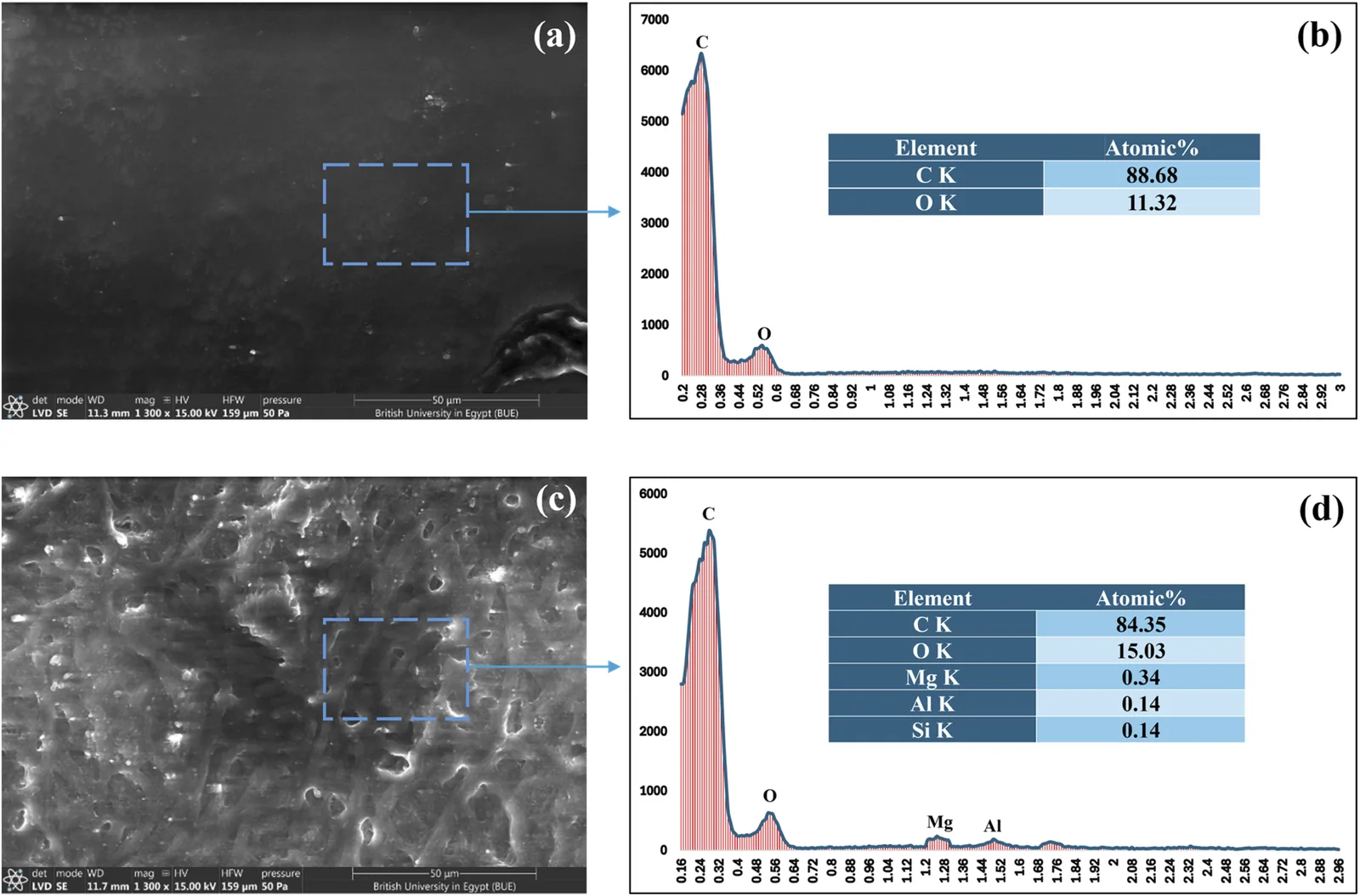
SEM/EDX characterization of neat epoxy and the 5 wt% Mg/Al-LDH/epoxy nanocomposite: (a and b) SEM micrograph and EDX spectrum of neat epoxy, respectively; (c and d) SEM micrograph and EDX spectrum of the nanocomposite, respectively.

The SEM image and EDX spectrum of the neat epoxy are presented in Fig. 9(a and b) respectively. The SEM micrograph exhibits a smooth and homogeneous surface morphology with no observable inorganic inclusions, indicating the absence of filler particles or structural irregularities. A few minor bright regions appeared, which were attributed to surface artifacts generated during specimen preparation. This uniform morphology confirmed the purity and structural consistency of the epoxy matrix, establishing a clear reference for subsequent nanocomposite analyses. The corresponding EDX spectrum displays only the characteristic carbon and oxygen peaks of the epoxy resin, with no additional elemental signals, thereby validating the chemical purity and the absence of contaminants or foreign phases in the unfilled epoxy.^59^

The surface morphology of hydrotalcite/epoxy nanocomposite, shown in Fig. 9(c), reveals a relatively homogeneous dispersion of the LDH nanosheets within the epoxy matrix together with small, localized agglomerates. The nanosheets appeared as bright regions embedded within the polymeric background, indicating good wetting and interfacial adhesion between the filler and matrix. The absence of significant cracks or phase separation suggests that the mixing and curing processes were well-optimized, leading to a uniform microstructure capable of efficient stress and charge transfer under electrical stress.

The microstructure examined is not entirely agglomeration-free, although the observed clustering is less pronounced than that at 7 wt% discussed below. The presence of these localized agglomerates is compatible with the measured maximum in breakdown strength at 5 wt%, suggesting that their adverse effects do not outweigh the beneficial effects of filler incorporation at this loading. However, this interpretation remains qualitative: the micrograph represents a limited observation region and does not directly establish interfacial adhesion strength, charge-transfer behavior, or nanoscale dispersion throughout the specimen.

The corresponding EDX spectrum in Fig. 9(d) confirms the presence of Mg and Al species within the epoxy matrix, alongside the dominant C and O peaks associated with the organic polymer backbone. Owing to the relatively low filler loading (5 wt%) and the carbon-rich nature of the epoxy matrix, Mg and Al are detected as low-intensity peaks with atomic fractions of 0.34 at% and 0.14 at%, respectively. The appearance of these characteristic elemental signals provides additional evidence for the successful incorporation of Mg/Al-LDH nanofillers into the epoxy host. Furthermore, the detection of a Si signal (0.14 at%) is consistent with the presence of silane-containing functional groups introduced during the surface functionalization process, supporting the FTIR results and further confirming the successful functionalization of the LDH nanoparticles. Additional SEM micrographs from different regions have been included in Fig. S8.

Elemental mapping was performed to examine the spatial distribution of the filler-associated elements within the selected region of the 5 wt% nanocomposite, as shown in Fig. 10. The EDX maps reveal a homogeneous distribution of the constituent elements throughout the examined region, with carbon and oxygen uniformly dispersed as the dominant elements of the epoxy matrix. More importantly, Mg and Al are distributed across the examined region, confirming the successful incorporation of Mg/Al-LDH within the composite. The detected Si signal is attributed to the APTES surface treatment, further supporting the presence of the silane-functionalized LDH phase. Importantly, the Mg, Al, and Si maps do not show pronounced elemental-rich zones, indicating that severe filler agglomeration was avoided at the optimum loading. The corresponding overlay image confirms the relatively uniform dispersion of the functionalized Mg/Al-LDH within the epoxy matrix. This improved dispersion is expected to enhance filler–matrix interactions, increase the effective interfacial area, and contribute to the observed improvement in dielectric and breakdown performance.

**Fig. 10:**
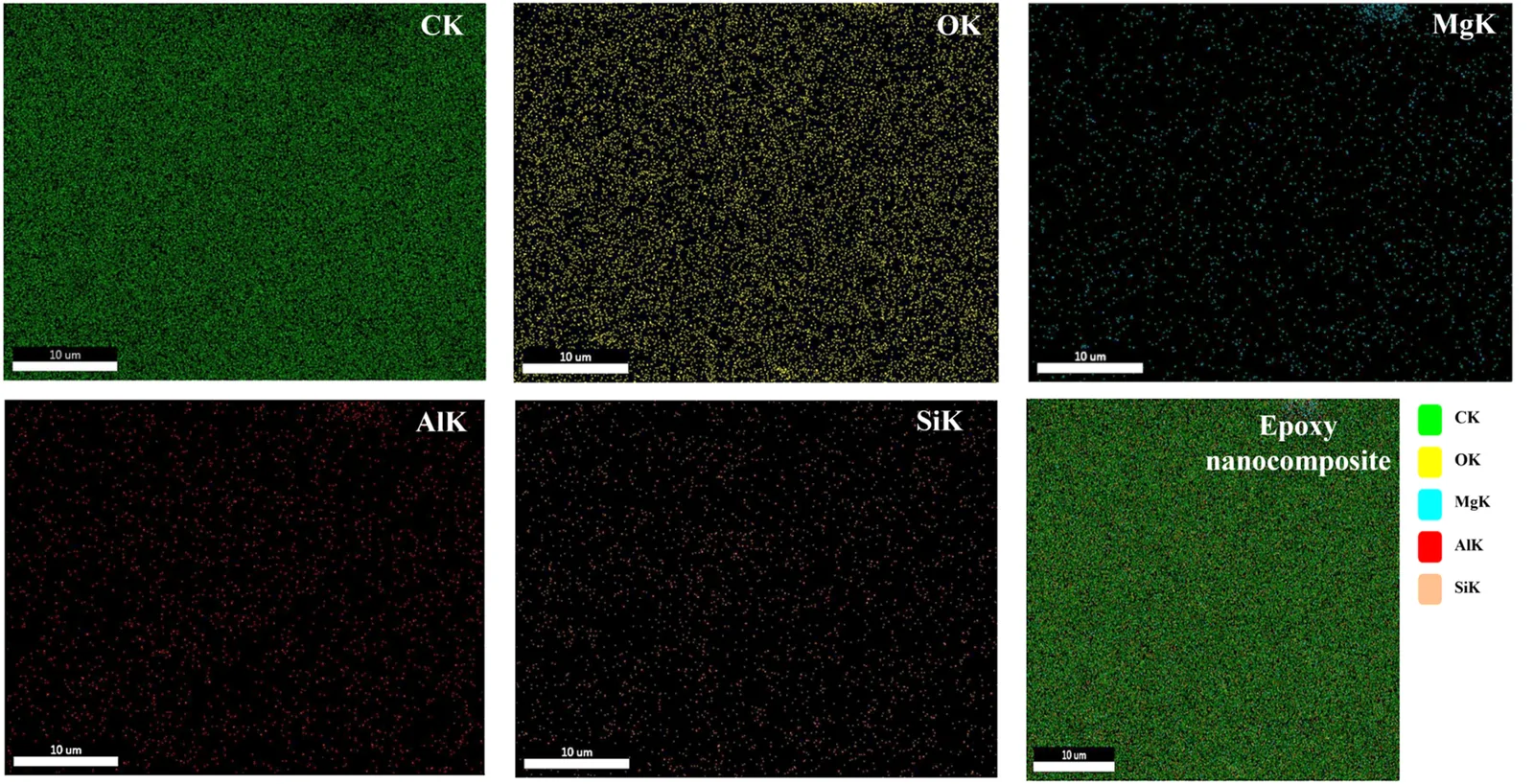
EDX elemental maps of the 5 wt% Mg/Al-LDH/epoxy nanocomposite.

To further support the interpretation of the performance deterioration at excessive filler loading, the dispersion of Mg/Al-LDH within the epoxy matrix was compared at 5 and 7 wt% using SEM imaging and semi-quantitative image analysis. Fig. 11 presents the SEM micrographs with red overlays highlighting the segmented agglomerated regions, while Table 4 summarizes the corresponding agglomeration descriptors. Compared with the 5 wt% nanocomposite, the 7 wt% sample exhibits more extensive localized agglomerated domains and a less homogeneous morphology. Increasing the filler loading from 5 to 7 wt% increased the agglomerated area fraction from 8.8% to 21.0%, while the agglomerate density decreased from 27.1 to 14.7 domains per 1000 µm^2^. Meanwhile, the D90 equivalent diameter increased from 2.84 to 3.31 µm, and the mean equivalent diameter increased from 1.41 to 1.78 µm. These results indicate that the increased loading does not produce a uniform increase in agglomerate size; instead, the segmented regions occupy a greater proportion of the observed area, with a larger upper-tail equivalent diameter. Together, the morphological observations and extracted descriptors support poorer filler dispersion at 7 wt% within the examined regions. Such clustering may reduce the effective filler–matrix interfacial area and promote local electric-field enhancement and defect-assisted conduction, providing a microstructural explanation for the deterioration in electrical insulation performance beyond 5 wt%.

**Fig. 11:**
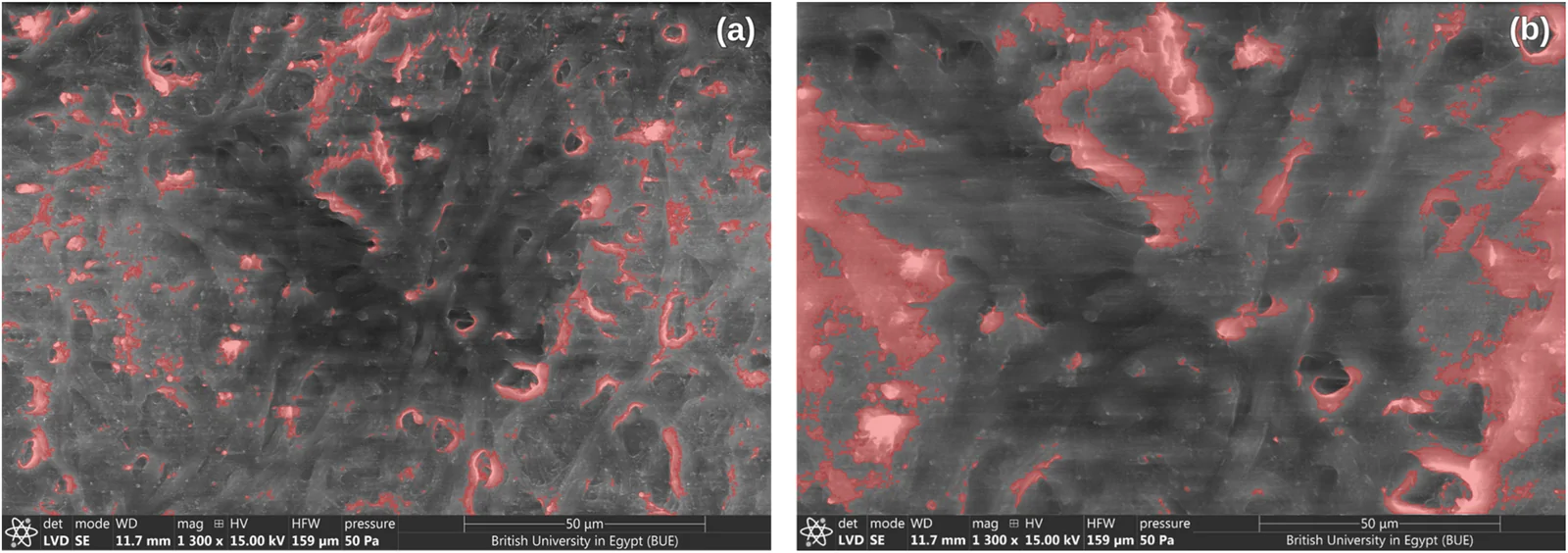
SEM micrographs of Mg/Al-LDH/epoxy nanocomposites containing (a) 5 wt% and (b) 7 wt% filler, with red overlays highlighting the segmented agglomerated regions.

**Table 4:** Quantitative agglomeration descriptors extracted from SEM micrographs of Mg/Al-LDH/epoxy nanocomposites at filler loadings of 5 and 7 wt%

Parameter	5 wt% Mg/Al-LDH/epoxy nanocomposite	7 wt% Mg/Al-LDH/epoxy nanocomposite
Agglomerated area fraction (%)	8.8	21
Agglomerate density (per 1000 µm^2^)	27.1	14.7
Mean equivalent diameter (µm)	1.41	1.78
D90 equivalent diameter (µm)	2.84	3.31

#### 3.5.2 Thermal characterization

The thermal stability of the neat epoxy and the optimized hydrotalcite/epoxy nanocomposite was evaluated using TGA, and the corresponding thermograms are shown in Fig. 12(a). The neat epoxy exhibited an onset degradation temperature (DTG-derived onset temperature) of 341 °C, whereas the incorporation of 5 wt% hydrotalcite shifted the onset to 357 °C, corresponding to an improvement of approximately 16 °C. In addition, the residual mass at 700 °C increased from 14.0% for neat epoxy to 18.1% for the hydrotalcite/epoxy nanocomposite.

**Fig. 12:**
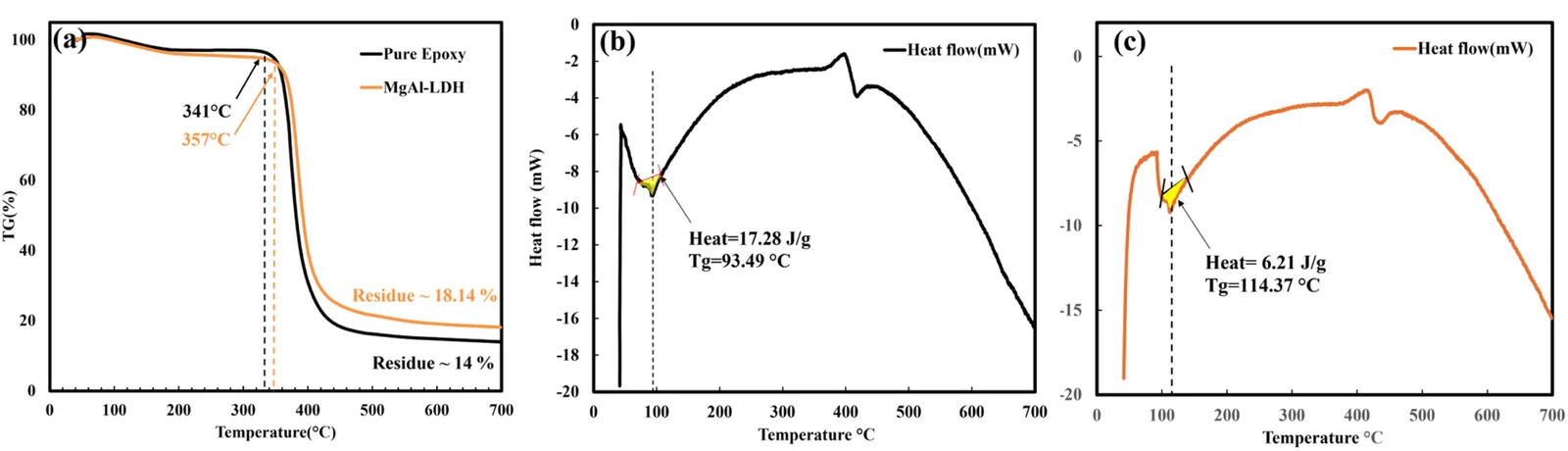
(a) TGA curves of neat epoxy and the 5 wt% Mg/Al-LDH/epoxy nanocomposite; (b) DSC thermogram of neat epoxy; (c) DSC thermogram of the 5 wt% Mg/Al-LDH/epoxy nanocomposite, respectively.

This improvement in thermal stability is attributed to the barrier effect of the layered hydrotalcite structure, which restricts heat transfer and slows the diffusion of volatile degradation products during thermal decomposition. However, the composite residue includes both polymer-derived char and filler-derived residue; therefore, this increase alone does not establish enhanced char formation or stabilization.^60,61^

The glass transition behavior of the composites was further evaluated using DSC, as shown in Fig. 12(b and c). Neat epoxy exhibited a glass transition temperature (*T*_g_) of 93.49 °C with a relaxation enthalpy of 17.28 J g^−1^. After hydrotalcite incorporation, *T*_g_ was increased markedly to 114.37 °C, whereas the relaxation enthalpy decreased to 6.21 J g^−1^.

The increase in *T*_g_ indicated the restricted segmental mobility of the epoxy chains owing to the presence of hydrotalcite platelets and the formation of constrained interfacial regions. These interfacial regions likely reduce the local free volume and suppress cooperative chain motion, thereby increasing the thermal resistance of the polymer network. The marked reduction in the relaxation enthalpy further supports the formation of a more constrained interphase, in which polymer chain mobility becomes partially immobilized by polymer–filler interactions.^62,63^ Second-heating measurements was not performed, so it is acknowledged that the contributions of thermal history and possible residual curing cannot be separately resolved from the present data.

From an insulation engineering perspective, this thermal improvement is particularly important. According to IEC 62271-1, the conductor temperatures in contact with the spacer materials may approach 105 °C during operation. While neat epoxy approaches this limit, the hydrotalcite/epoxy nanocomposite exhibits a substantially higher *T*_g_, suggesting potential for improved resistance to thermally induced softening relative to neat epoxy, and improved dimensional stability for GIS/GITL insulation services.^64^

Revisiting the objectives of this study, the results indicate improvements in selected breakdown and thermal properties under the investigated experimental conditions. At 50 Hz, the relative permittivity ranged from 4.01 to 5.08 across the investigated formulations, while the dielectric loss tangent remained below 0.01 despite increasing with filler incorporation. Among the tested loadings, the 5 wt% nanocomposite exhibited the highest mean AC breakdown strength of 36.75 kV mm^−1^, representing a 22.2% increase over neat epoxy, with a Weibull characteristic breakdown strength of 37.29 kV mm^−1^. This formulation also showed an increase in degradation onset temperature from 341 to 357 °C and in glass transition temperature from 93.49 to 114.37 °C. The higher residual mass cannot independently establish enhanced polymer char formation because it includes filler-derived residue. These findings support further investigation of APTES-functionalized Mg/Al-LDH as a modifier for epoxy insulation; however, long-term aging and electrothermal testing under representative GIS/GITL service conditions are needed to assess its suitability for practical application.

#### 3.5.3 Compression test

Compression testing was performed according to ASTM D695 to evaluate the mechanical response of the neat epoxy and Mg/Al-LDH epoxy composite at its optimum loading under compressive loading. Five specimens were tested for each formulation, and the obtained compressive stress values are summarized in Table 5.

**Table 5:** Maximum compressive stress of neat epoxy and the 5 wt% Mg/Al-LDH/epoxy nanocomposite measured using five specimens per formulation according to ASTM D695

Reading	Compressive stress (MPa)	
	Pure epoxy	Epoxy with 5 wt% Mg/Al-LDH nanocomposite
1	103.94	129.92
2	107.48	129.53
3	100.39	127.95
4	102.76	127.17
5	105.12	129.13
Mean ± std deviation	103.94 ± 2.64	128.74 ± 1.15

The neat epoxy exhibited an average maximum compressive stress of 103.9 MPa, whereas the Mg/Al-LDH/epoxy composite reached 128.7 MPa, corresponding to an improvement of approximately 23.9%. The difference in mean maximum recorded compressive stress was statistically significant (Welch's *t*-test: *t* = 19.26, *d*_f_ = 5.45, *p* < 0.001), with a mean difference of 24.80 MPa and a 95% confidence interval of 21.57–28.03 MPa.

This enhancement indicates that the incorporation of Mg/Al-LDH improved the load-bearing capability of the epoxy matrix under compression. The improvement can be attributed to the reinforcing effect of the rigid LDH nanosheets, which can restrict localized matrix deformation and promote more efficient stress transfer between the epoxy matrix and the inorganic filler phase.^65,66^ In addition, the lamellar structure of LDH particles can provide a relatively large interfacial area, allowing the applied compressive load to be more effectively distributed within the composite.^65^

The post-compression photographs further reveal a clear difference in the failure morphology of the two systems as shown in Fig. 13. The neat epoxy specimen mainly exhibited deformation/barrelling without complete macroscopic fracture, indicating a deformation-dominated compressive response. According to ASTM D695, when a specimen does not fail by a distinct fracture and instead continues to flatten or deform, the reported compressive strength should be interpreted carefully because it may depend on the selected deformation or maximum-load criterion.^29^ Therefore, the value reported for neat epoxy in this work is more appropriately regarded as the maximum compressive stress recorded during the test. In contrast, the Mg/Al-LDH epoxy nanocomposite showed visible cracking after reaching a higher compressive stress. This observation does not contradict the improved compressive performance; rather, it suggests that Mg/Al-LDH increased the resistance of the epoxy matrix to compressive deformation, while the final failure occurred more abruptly after the critical stress level was exceeded. Such crack-assisted failure may be associated with local stress concentration at filler/matrix interfaces, possible LDH agglomerates, or small casting defects, which can act as crack-initiation sites during the final stage of loading.^67^

**Fig. 13:**
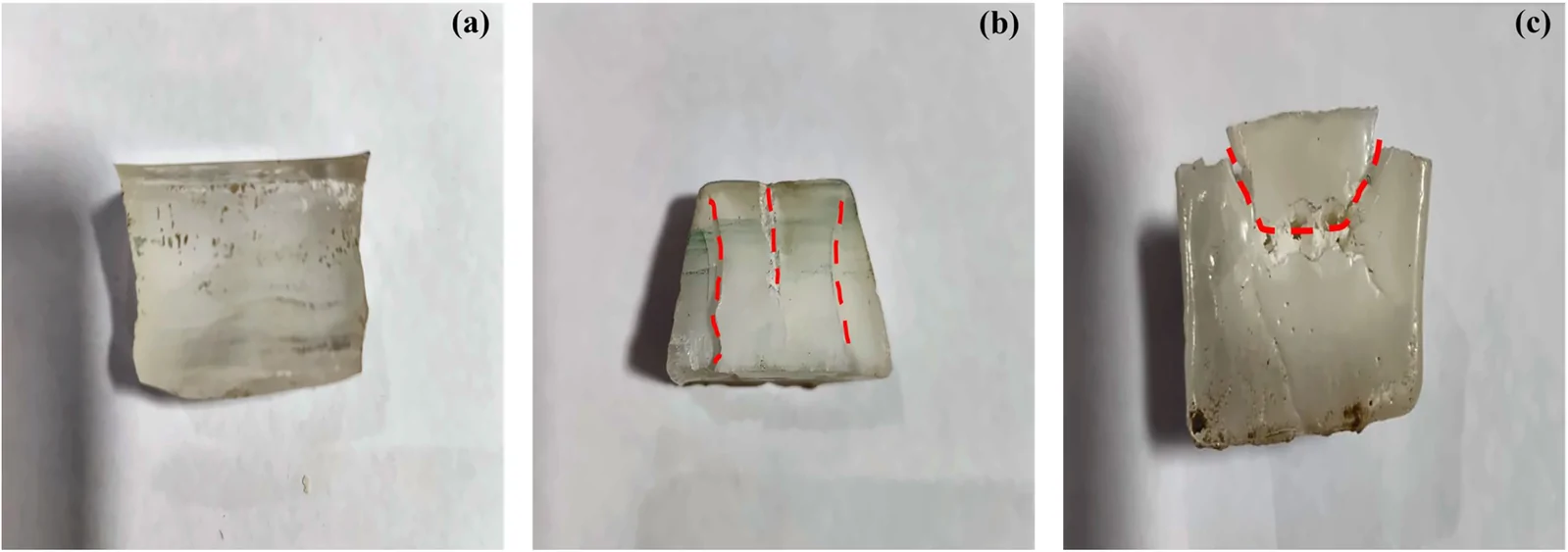
Post-compression morphology of: (a) neat epoxy and (b and c) the 5 wt% Mg/Al-LDH/epoxy nanocomposite.

## 4 Conclusion

Mesoporous Mg/Al hydrotalcite nanoparticles with layered platelet morphology were successfully synthesized, APTES-functionalized, and incorporated into epoxy resin to develop nanocomposites for high-voltage GIS/GITL solid insulation. The prepared composites were systematically evaluated through dielectric spectroscopy, AC breakdown testing, Weibull reliability analysis, and structural and thermal characterization to identify the optimum filler loading and clarify the dominant enhancement mechanisms. The incorporation of Mg/Al-LDH maintained a relatively moderate permittivity variation and a dielectric loss tangent below 0.01 at 50 Hz across the investigated filler loadings. The optimum electro-insulation performance was achieved at 5 wt%, where the nanocomposite exhibited an AC breakdown strength of 36.75 kV mm^−1^, corresponding to a 22.2% improvement over neat epoxy, together with enhanced Weibull reliability.

Morphological observations showed limited localized agglomeration at 5 wt% and more pronounced clustering at 7 wt%, consistent with a balance between beneficial filler effects and increasing microstructural heterogeneity. Thermal analysis further demonstrated improved thermal stability, including an increase in degradation onset temperature from 341 °C to 357 °C and an increase in glass transition temperature to 114.37 °C. Furthermore, preliminary compression testing yielded a maximum recorded compressive stress of 128.7 MPa for the 5 wt% Mg/Al-LDH/epoxy nanocomposite, compared with 103.9 MPa for neat epoxy, corresponding to an increase of approximately 23.9%. However, the different deformation and cracking responses and the absence of strain measurements limit conclusions regarding comparative compressive strength and mechanical reliability.

Among the investigated formulations, the 5 wt% Mg/Al-LDH/epoxy nanocomposite exhibited the highest mean AC breakdown strength, with a dielectric loss tangent below 0.01 at 50 Hz. Compared with neat epoxy, this formulation also showed higher degradation onset and glass transition temperatures, and a higher maximum recorded compressive stress. These findings demonstrate that APTES-functionalized mesoporous Mg/Al-LDH is a promising nanofiller for high-performance epoxy insulation in GIS/GITL applications. The identified optimum of 5 wt% is limited to the investigated loadings and measured mean AC breakdown strength. The study is also limited by qualitative dispersion assessment, indirect interpretation of charge-trapping mechanisms, and testing under long-term aging and electrothermal cycling tests under relevant GIS/GITL service conditions, so future work should address these limitations.

## Author contributions

Mahmoud Ezzat: methodology, formal analysis, investigation, data curation, writing – original draft, visualization. Abdelrahman Said: conceptualization, supervision, validation, writing – review & editing. Musa A. Said: supervision, resources, validation. Hany M. Abd El-Lateef: supervision, resources, validation. Mousa A. Abd-Allah: conceptualization, supervision, resources, validation, writing – review & editing. S. M. A. El-Gamal: conceptualization, supervision, resources, validation, writing – review & editing. M. Ramadan: conceptualization, methodology, formal analysis, writing – original draft& review, supervision.

## Conflicts of interest

The authors declare that they have no known competing financial interests or personal relationships that could have appeared to influence the work reported in this paper.

## Supplementary Material

RA-016-D6RA07272K-s001

RA-016-D6RA07272K-s002

## Data Availability

Data will be made available on reasonable request. Supplementary information (SI) is available. See DOI: https://doi.org/10.1039/d6ra07272k.

## References

[cit1] Ramadan M., Ezzat M., Abd-Allah M. A., El-Gamal S. M. A., Said A. (2026). Sci. Rep..

[cit2] Said A., Ezzat M., Abd-Allah M. A., El-Gamal S. M. A., Ramadan M. (2026). Electr. Power Syst. Res..

[cit3] Alsoud A., Daradkeh S. I., Al-Bashaish S. R., Shaheen A. A., Jaber A. M. D., Abuamr A. M., Mousa M. S., Holcman V. (2024). Polymers.

[cit4] Xing Y., Wang Z., Liu L., Xu Y., Yang Y., Liu S., Zhou F., He S., Li C. (2022). High Voltage.

[cit5] Hari R., Mohana Rao M. (2022). Int. J. Emerging Electr. Power Syst..

[cit6] Wang S., Chen Y., Liu C., Yao K., Mao J., Cheng Y., Wang Z., Gong R. (2024). IEEE Trans. Dielectr. Electr. Insul..

[cit7] Al Soud A., Daradkeh S. I., Knápek A., Holcman V., Sobola D. (2023). Phys. Scr..

[cit8] Jeong S. H., Bin Song J., Choi Y. H., Kim S. G., Go B. S., Park M., Lee H. (2016). Composites, Part B.

[cit9] Fuqiang T., Lin Z., Junliang Z., Xiao P. (2017). Composites, Part B.

[cit10] Sukesh Babu M., Sarathi R., Jayantilal Vasa N., Imai T. (2020). High Voltage.

[cit11] Sundar U., Lao Z., Cook-Chennault K. (2020). Polymers.

[cit12] Adnan M. M., Tveten E. G., Glaum J., Ese M. H. G., Hvidsten S., Glomm W., Einarsrud M. A. (2019). Adv. Electron. Mater..

[cit13] Al Soud A. , Daradkeh S., Knápek A., Liedermann K., Holcman V. and Sobola D., in Proceedings II of the Conference Student EEICT, Brno University of Technology, 2023, vol. 2, pp. 253–257

[cit14] Ma W. X., Feng C., Li W., Du X. Y., Liu J. D., Zhao Z. B. (2026). Appl. Surf. Sci..

[cit15] Zhao Z. B., Liu J. D., Du X. Y., Wang Z. Y., Zhang C., Ming S. F. (2022). Colloids Surf., A.

[cit16] Wang Z. Y., Sun X., Wang Y., Liu J. D., Zhang C., Zhao Z. B., Du X. Y. (2023). Ceram. Int..

[cit17] Frunza L. , Frunza S., Ganea C. P., Zgura I., Neatu F. and Parvulescu V. I., Dielectric Properties of LDH-type Layered Materials Containing Zn or Mg Ions: on the Non Monotonous Temperature Dependence of Relaxation Times, 2015, vol. 18

[cit18] Ye T., Li H., Du M., Ma X., Liu X., Wen L. (2021). RSC Adv..

[cit19] Ritu, Patel M. K., Gupta M. K. (2024). J. Vinyl Addit. Technol..

[cit20] Abdul Razak N. I., Yusoff N. I. S. M., Ahmad M. H., Zulkifli M., Wahit M. U. (2023). Polymers.

[cit21] Kilikevičius S., Mishnaevsky L., Zeleniakiene D. (2025). Comput. Mater. Sci..

[cit22] Asaad J., Gomaa E., Bishay I. K. (2008). Mater. Sci. Eng., A.

[cit23] ASTM International , ASTM D150-18: Standard Test Methods for AC Loss Characteristics and Permittivity (Dielectric Constant) of Solid Electrical Insulation, 2018, 10.1520/D0150-18

[cit24] ASTM International , ASTM D257-14(2021)e1: Standard Test Methods for DC Resistance or Conductance of Insulating Materials, 2021, 10.1520/D0257-14R21E01

[cit25] ASTM International , ASTM D495-22: Standard Test Method for High-Voltage, Low-Current Dry Arc Resistance of Solid Electrical Insulation, 2022, 10.1520/D0495-22

[cit26] Said A., Abd-Allah M. A., Nawar A. G., Elsayed A. E., Kamel S. (2022). J. Mater. Sci.: Mater. Electron..

[cit27] Ezzat M., Said A., Abd-Allah M. A., El-Gamal S. M. A., Ramadan M. (2026). J. Mater. Sci.: Mater. Electron..

[cit28] ASTM International , ASTM D149-20: Standard Test Method for Dielectric Breakdown Voltage and Dielectric Strength of Solid Electrical Insulating Materials at Commercial Power Frequencies, 2020, 10.1520/D0149-20

[cit29] ASTM International , ASTM D695-26: Standard Test Method for Compressive Properties of Rigid Plastics, 2026, 10.1520/D0695-26

[cit30] Karlsson M. E., Xu X., Hillborg H., Ström V., Hedenqvist M. S., Nilsson F., Olsson R. T. (2020). RSC Adv..

[cit31] Tsen A. W., Brown L., Levendorf M. P., Ghahari F., Huang P. Y., Havener R. W., Ruiz-Vargas C. S., Muller D. A., Kim P., Park J. (2012). Science.

[cit32] Cheng L., Ma H., Liu X., Tang Q., Yang K., Zhang Y., Zhang H., Wang H., Xiong S. (2025). Chem. Eng. J..

[cit33] Abd El-Lateef H. M., Khalaf M. M., Ramadan M., Mohsen A. (2026). J. Mater. Res. Technol..

[cit34] Wiyantoko B., Kurniawati P., Purbaningtias T. E., Fatimah I. (2015). Procedia Chem..

[cit35] Aminifazl A., Karunarathne D. J., Golden T. D. (2024). Molecules.

[cit36] Wu J., Peng D., He Y., Du X., Zhang Z., Zhang B., Li X., Huang Y. (2017). Materials.

[cit37] Ryu I. S., Liu X., Jin Y., Sun J., Lee Y. J. (2018). RSC Adv..

[cit38] Sardon H., Irusta L., Santamaría P., Fernández-Berridi M. J. (2012). J. Polym. Res..

[cit39] Mohsen A., Kohail M., Alharbi Y. R., Abadel A. A., Soliman A. M., Ramadan M. (2023). Case Stud. Constr. Mater..

[cit40] Chhetri S., Chandra Adak N., Samanta P., Chandra Murmu N., Kuila T. (2018). Ind. Eng. Chem. Res..

[cit41] Tao Q., He H., Li T., Frost R. L., Zhang D., He Z. (2014). J. Solid State Chem..

[cit42] Mureseanu M., Eliescu A., Ignat E.-C., Carja G., Cioatera N. (2022). C. R. Chim..

[cit43] Li X., Wang G., Lu Y., Shi F., Ma Y., Li D. (2025). Arabian J. Chem..

[cit44] Wijaya A., Siregar P. M. S. B. N., Badri A. F., Palapa N. R., Amri A., Ahmad N., Lesbani A. (2023). Period. Polytech., Chem. Eng..

[cit45] Wang X., Pakdel A., Zhang J., Weng Q., Zhai T., Zhi C., Golberg D., Bando Y. (2012). Nanoscale Res. Lett..

[cit46] Mashkoor F., Shoeb M., Khan M. N., Jeong C. (2024). Polymers.

[cit47] Manjumol C. C., Limna Mol V. P., Deepa B., Sabukuttan D., Nevin K. G. (2025). Bionanoscience.

[cit48] Ramadan M., Amin M. S., Mohsen A. (2026). Sci. Rep..

[cit49] Xia X., Zhong Z., Weng G. J. (2017). Mech. Mater..

[cit50] Wang L., Yang J., Cheng W., Zou J., Zhao D. (2021). Front. Mater..

[cit51] What Is the Acceptable Value for a Tan Delta Test?, https://www.hvtesters.com/what-is-the-acceptable-value-for-a-tan-delta-test/, accessed 26 November 2025

[cit52] Röchling , Durostone® Tubes EPC 22, https://www.roechling.com/industrial/materials/composites/gfk/gfk-ep/durostone-tubes-epc-22-715238, accessed November 2025

[cit53] Lian H., Yan J., Xie J., Song Y., Zhou H., Kang Y., Fangcheng L., Xie Q. (2020). Polym. Compos..

[cit54] Zhang H., Zhang Z., Liu C., Jiang X., Hu J., Hu Q. (2025). Polymer.

[cit55] Shengtao L., Mingru L. (2024). iEnergy.

[cit56] Szymoniak P., Qu X., Abbasi M., Pauw B. R., Henning S., Li Z., Wang D.-Y., Schick C., Saalwächter K., Schönhals A. (2021). Soft Matter.

[cit57] Lu H., Nutt S. (2003). Macromolecules.

[cit58] Jia B., Zhou J., Chen J., Zhang Z., Wang Y., Lv Z., Wu K. (2023). Nanomaterials.

[cit59] Li Q., Peng X., Zhong X., Zhou Y., Luo T., Zhang X. (2024). Polym. Compos..

[cit60] Mohsen A., Amin M. S., Selim F. A., Ramadan M. (2024). Constr. Build. Mater..

[cit61] Ramadan M., Sayed D. G., Selim F. A. (2024). Constr. Build. Mater..

[cit62] Omar H., Smales G. J., Henning S., Li Z., Wang D. Y., Schönhals A., Szymoniak P. (2021). Polymers.

[cit63] Oner G., Nadaroglu H., Acar V., Gok S. (2023). Biomass Convers. Biorefin..

[cit64] Iderus S., Peter G., Vivekananda G. (2023). J. Eng..

[cit65] Logesh K., Bupesh Raja V. K. (2019). Int. J. Adv. Manuf. Technol..

[cit66] Li X., Wang Q., Cui X., Feng X., Teng F., Xu M., Su W., He J. (2022). Polymers.

[cit67] Zeinedini A., Shokrieh M. M. (2024). Appl. Phys. Rev..

