# Supplementary material for: Epoxy reinforced with mesoporous hydrotalcite for high-voltage insulation application
Source: RSC Adv. 2026 Sep 24;16(45):57214–31. doi: 10.1039/d6ra07272k (PMC13602962; doi:10.1039/d6ra07272k)
Supplement: Supplementary file 1 [file RA-016-D6RA07272K-s001.pdf]

Supplementary Information (SI) for RSC Advances.
This journal is © The Royal Society of Chemistry 2026
Supplementary Information (SI) for RSC Advances.
This journal is © The Royal Society of Chemistry

Supporting Information

Epoxy Reinforced with Mesoporous Hydrotalcite for High–Voltage Insulation Application

Table S1. List of the chemicals' specifications used in this study.

Material	IUPAC name	Purity (%)	Producer (City, Country)
Epoxy resin	Bisphenol-A diglycidyl ether (DGEBA)	—	Chemicals for Modern Building International, Giza, Egypt
Hardener	Polyamine curing agent	—	Chemicals for Modern Building International, Giza, Egypt
$Mg(NO_3)_2$	magnesium nitrate	98 %	Sigma-Aldrich, St. Louis, USA
Aluminum chloride ($AlCl_3$)	Aluminum chloride	99.999 %	Sigma-Aldrich, St. Louis, USA
Sodium carbonate (Na_2CO_3)	Sodium carbonate	≥ 99.5 %	Sigma-Aldrich, St. Louis, USA
Cetyltrimethylammonium bromide (CTAB)	Hexadecyltrimethylammonium bromide	≥ 99 %	Sigma-Aldrich, St. Louis, USA
Silane coupling agent	(3-Aminopropyl) triethoxysilane (APTES)	99 %	Sigma-Aldrich, St. Louis, USA
Ethanol	Ethanol	99 %	El Nasr Pharmaceutical Chemicals, Cairo, Egypt

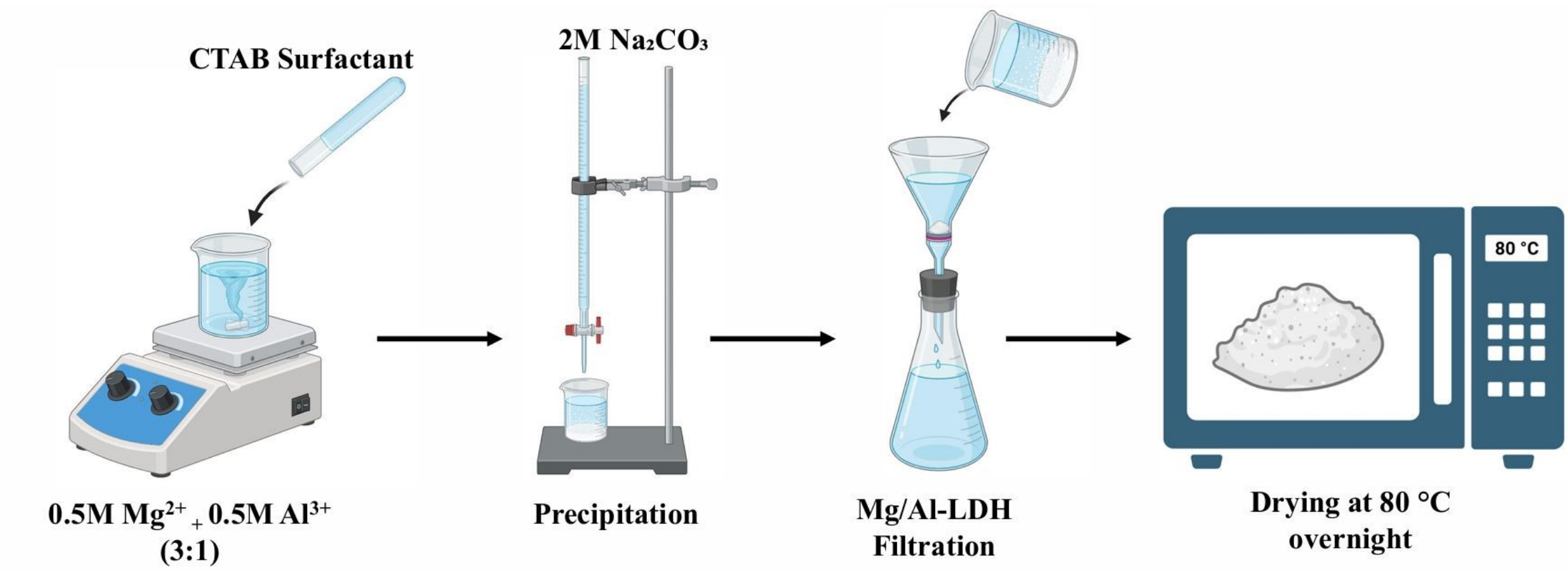

Fig. S1. Preparation of Mg/Al-LDH nanoparticles.

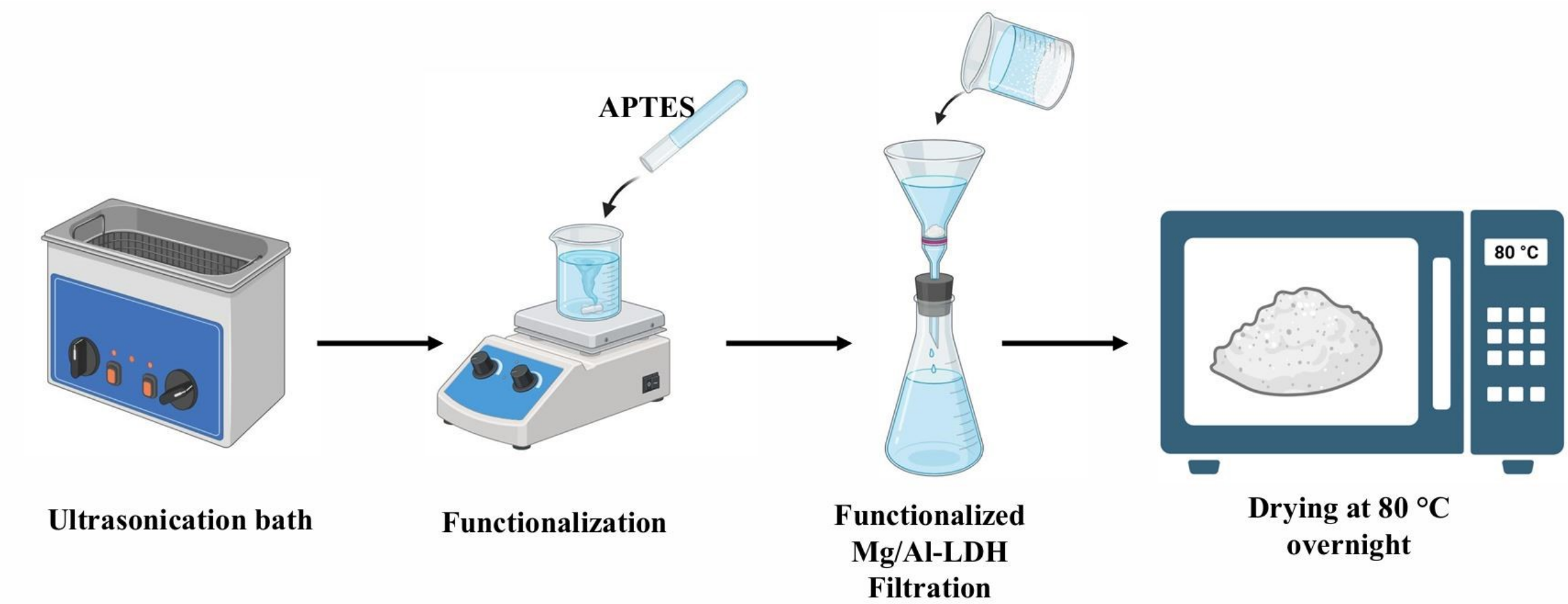

Fig. S2. Surface functionalization of Mg/Al-LDH nanoparticles.

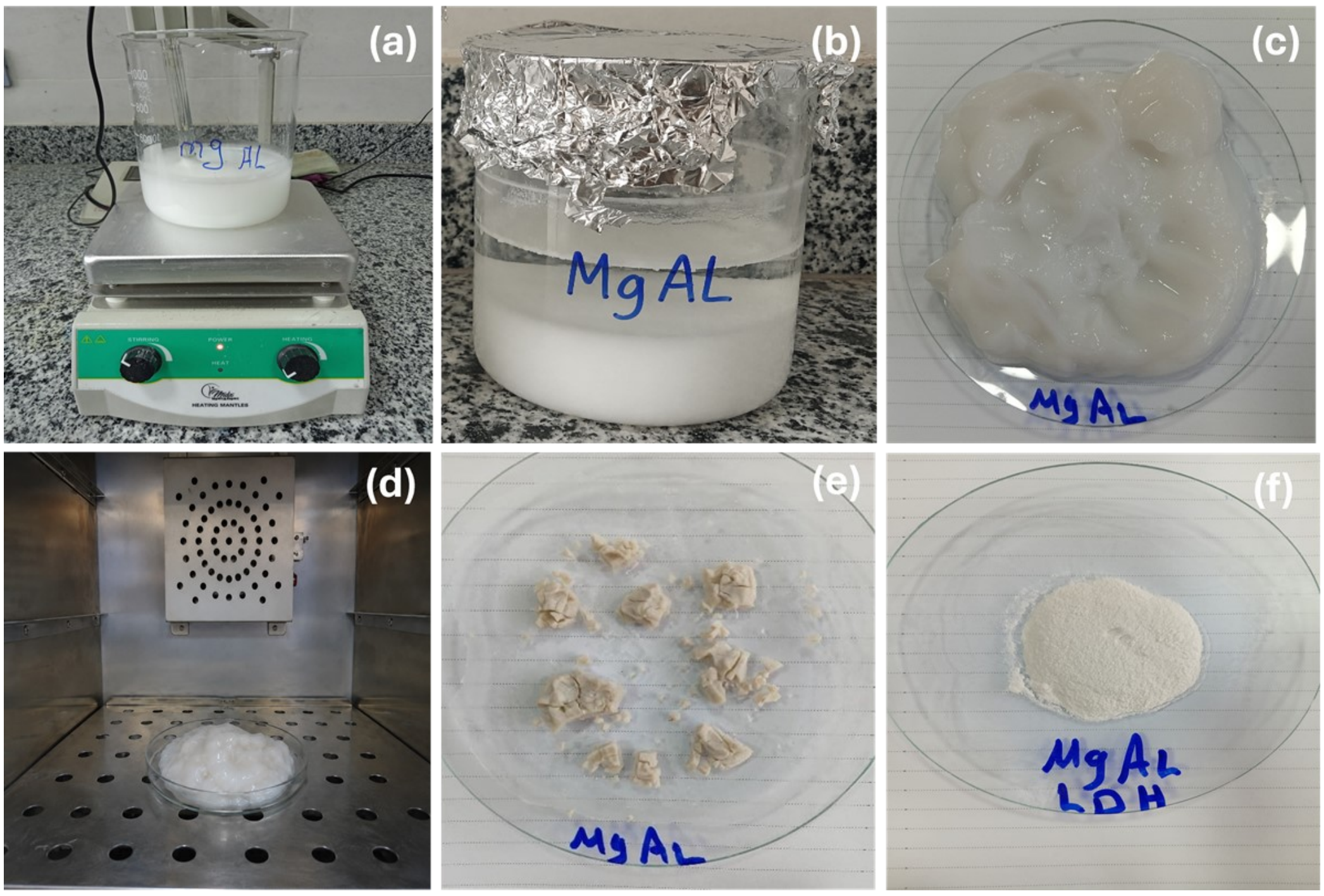

Fig. S3. Preparation steps of Mg/Al-LDH nanoparticles: (a) precursor mixing, (b) aging, (c) precipitate formation, (d) oven drying, (e) dried product, and (f) final Mg/Al-LDH powder.

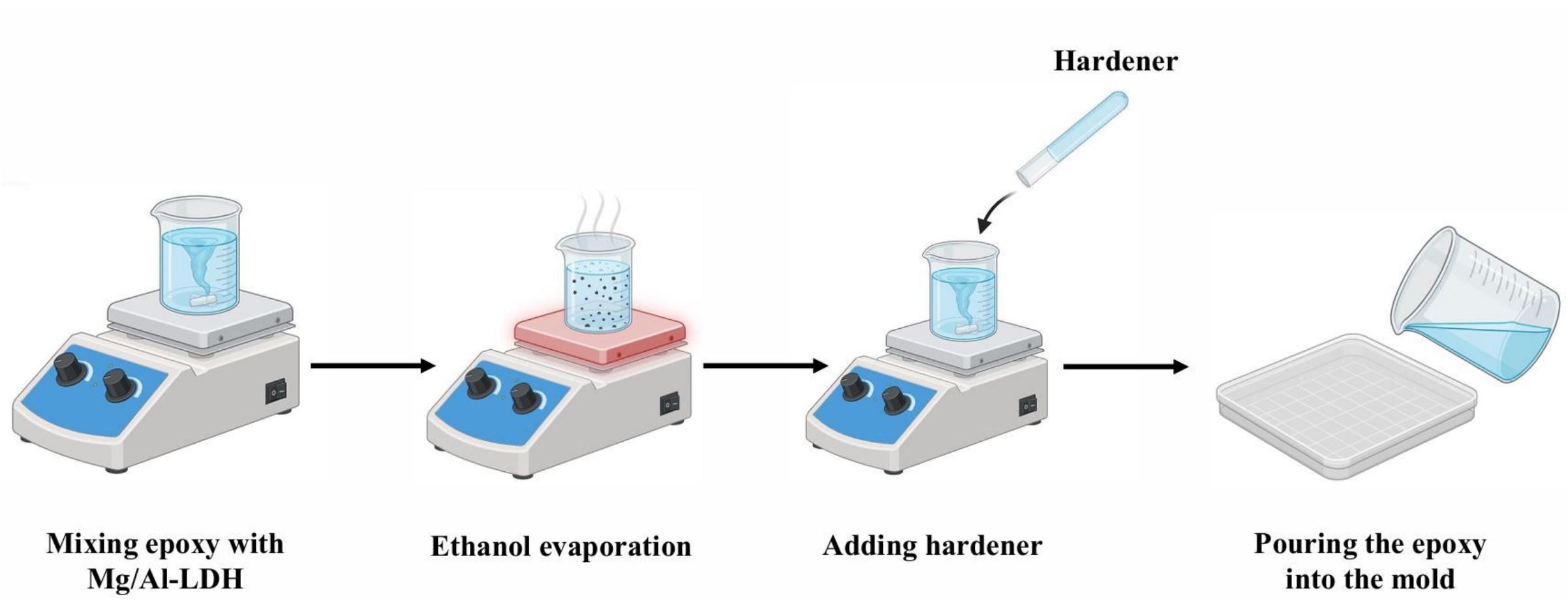

Fig. S4. Preparation of epoxy nanocomposite samples.

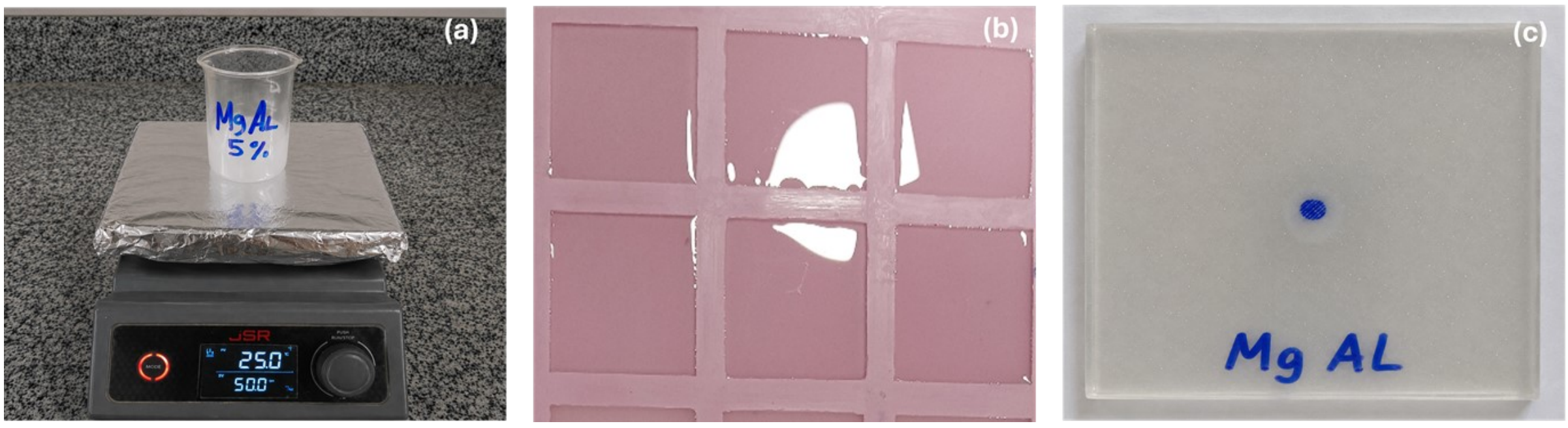

Fig. S5. Preparation of Mg/Al-LDH epoxy nanocomposite samples: (a) dispersion of Mg/Al-LDH in epoxy resin, (b) casting of the epoxy nanocomposite into molds, and (c) final cured Mg/Al-LDH epoxy sample.

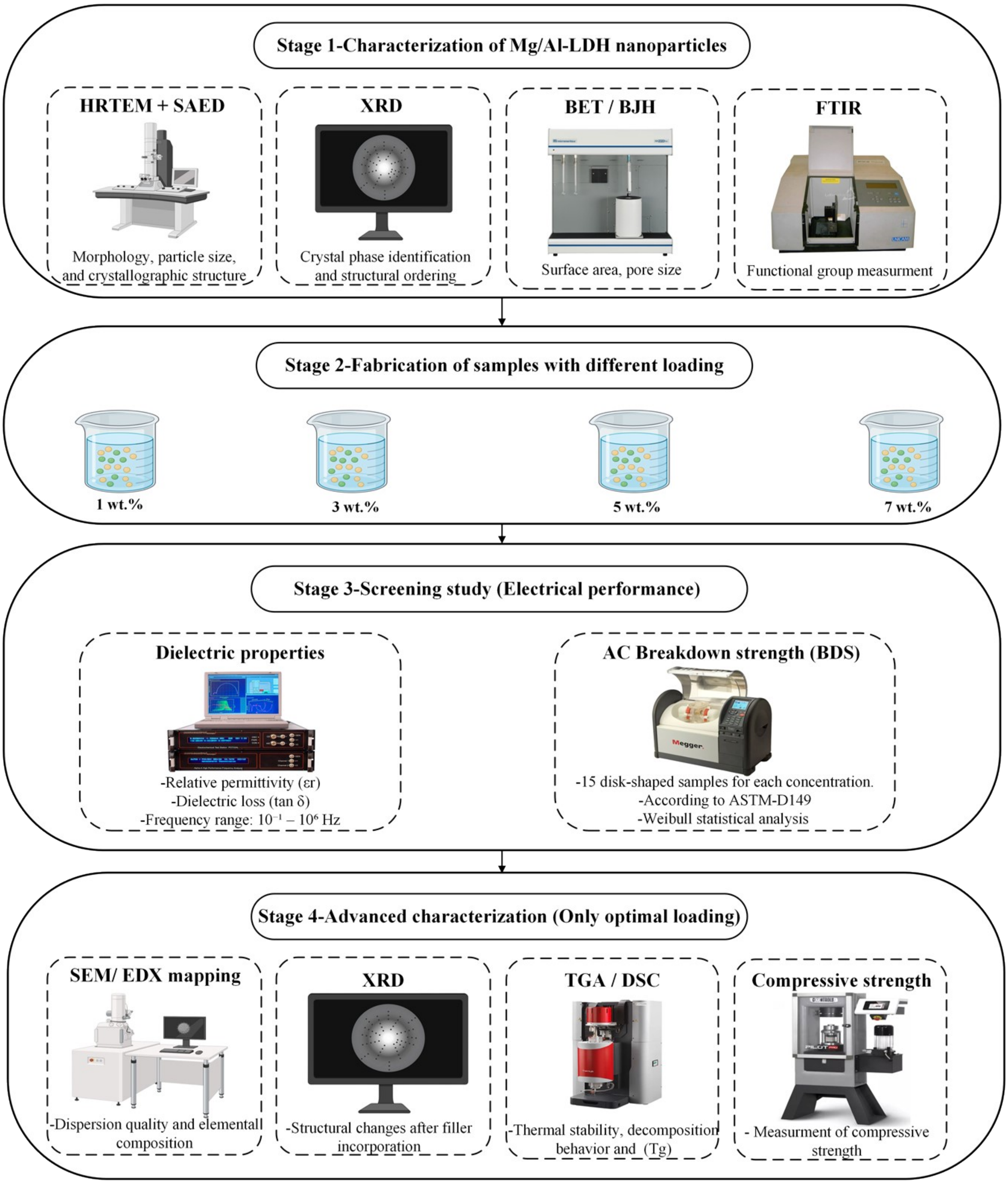

Fig. S6. Schematic illustration of experimental methodology.

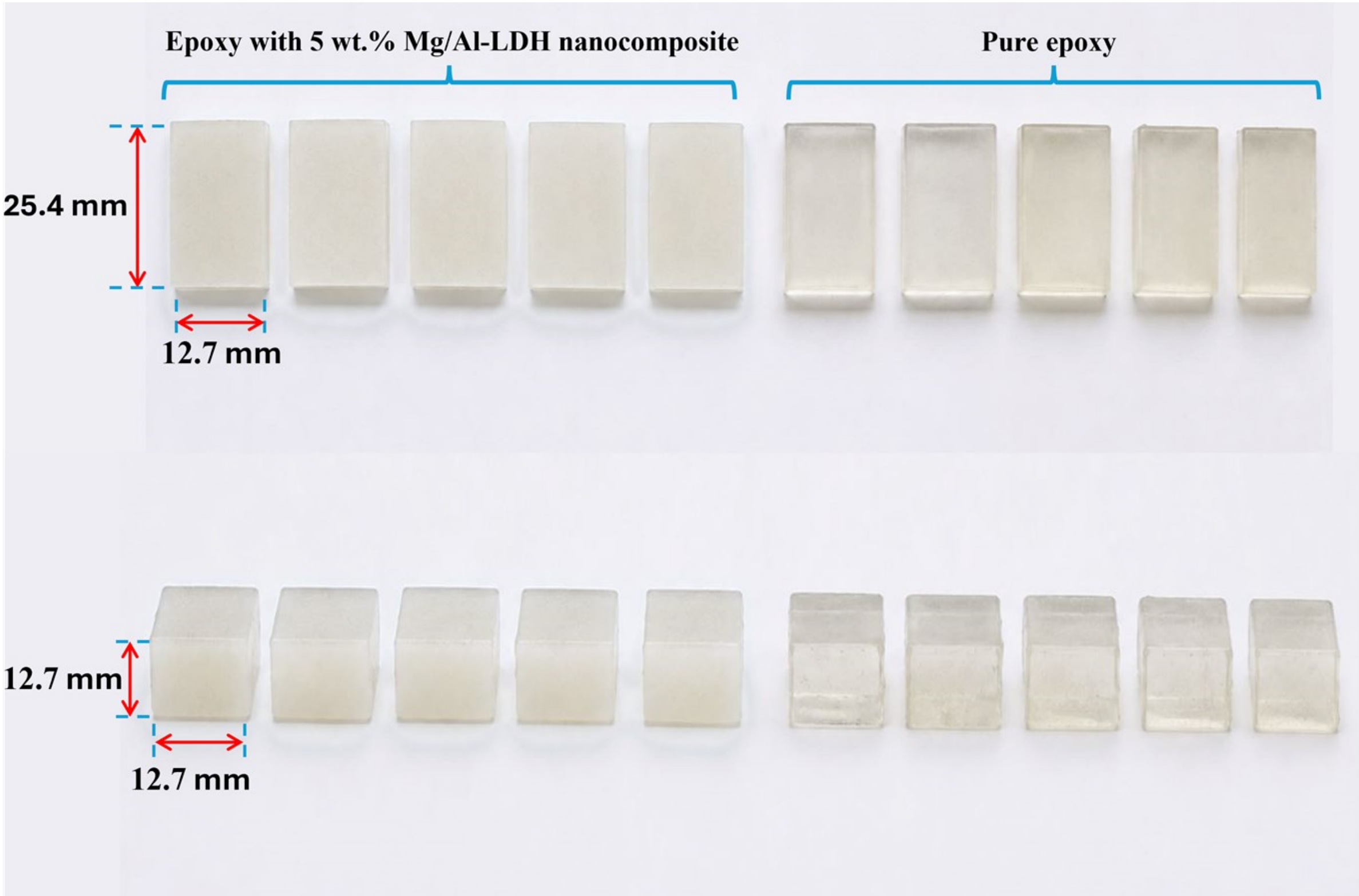

Fig. S7. compression test specimens of pure epoxy and epoxy filled with 5 wt.% Mg/Al-LDH nanocomposite before testing.

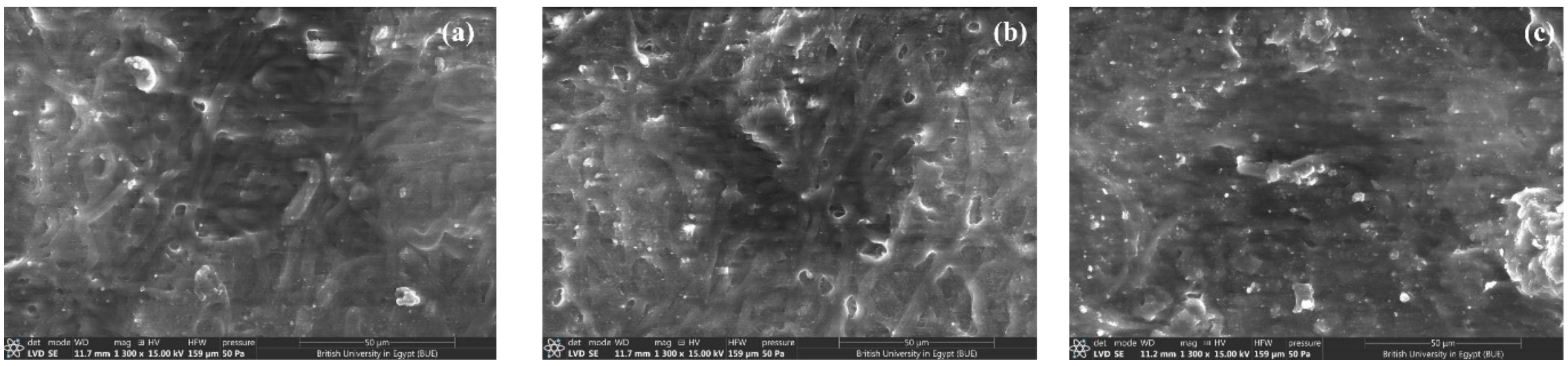

Fig. S8. Additional SEM micrographs acquired from different regions of Mg/Al-LDH/epoxy nanocomposites, illustrating spatial variations in filler dispersion and localized agglomeration.
